# Effects and mechanisms of microplastic types on plant uptake of per- and polyfluorinated alkyl substances

**DOI:** 10.1016/j.eehl.2026.100216

**Published:** 2026-01-19

**Authors:** Qian Gu, Pengfei Zhou, Yi Kong, Chenzhuo Song, Qian Zhang, Xinyi Cui

**Affiliations:** State Key Laboratory of Water Pollution Control and Green Resource Recycling, School of the Environment, Nanjing University, Nanjing 210023, China

**Keywords:** PFASs, Microplastics, Tire wear particles, Plant uptake

## Abstract

Microplastics (MPs) and per- and polyfluoroalkyl substances (PFASs) frequently co-occur in agricultural soils. MP type–dependent interactions with soil and plants can modify PFASs environmental behavior, complicating assessments of PFAS-related ecological and human exposure risks. This study investigated the effects of three MPs, namely polyvinyl chloride (PVC), polylactic acid (PLA), and tire wear particles (TWP), on the uptake of 10 PFASs by pak choi (*Brassica chinensis* L). PVC at 0.01%, 0.05%, and 0.5% significantly (*p* < 0.05) increased PFASs accumulation in shoots by 1.31–1.70 fold. The upregulation of aquaporin-related genes in pak choi co-exposed to PVC and PFASs represents a potential mechanism for the enhanced uptake and translocation of PFASs. In contrast, PLA did not affect PFASs accumulation but inhibited plant growth by downregulating lipid and amino acid metabolism. TWP at 0.01%, 0.05%, and 0.5% significantly (*p* < 0.05) decreased PFASs uptake in shoots by 37.4%–54.1%, primarily through its inhibitory effects on plant growth (9.2%–16.3% decrease in biomass) and transpiration rate (reduced to 73% of the control). The phytotoxicity of TWP was confirmed by metabolomic profiling, which was associated with downregulation of key lipid, amino acid, and jasmonic acid–related metabolites. This work provides the first systematic comparison of the effects of PVC, PLA, and TWP on PFASs uptake in vegetables, integrating multi-omics analyses to uncover mechanisms and distinct MP type-dependent effects on PFASs bioaccumulation. These findings highlight complex interactions between MPs and PFASs in soil and underscore the need to assess co-contaminant risks by MP types.

## Highlights

• MPs and PFASs widely co-exist in soils.
• Effects of three MPs on the uptake of 10 PFASs by pak choi were investigated.
• PVC increased PFAS uptake through upregulating aquaporin-related genes expression.
• TWP reduced PFAS uptake due to its phytotoxicity based on metabolomic profiling.

## Graphical abstract

**Figure:**
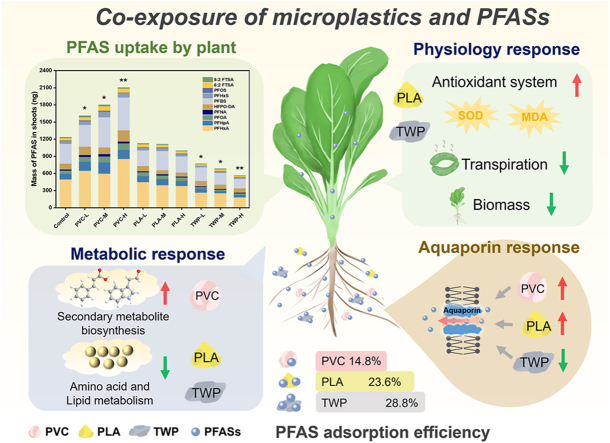

## 1 Introduction

Per- and polyfluorinated alkyl substances (PFASs) are widely applied across industrial and consumer sectors because of their distinctive physical and chemical characteristics [1]. Their persistence has led to frequent detection in agricultural soils, with concentrations reaching up to 641 ng/g [2]. PFASs in soil can be taken up by vegetables and crops, subsequently entering the human body through the food chain. For example, in Hubei Province, China, leafy vegetables emerged as the dominant dietary pathway for human exposure to PFASs, contributing more than 60% to the total body burden [3]. The accumulation of PFASs in edible plants represents a direct route of dietary exposure, with documented toxicological impacts on human liver, kidney, nervous system, and immune system [4].

Plant uptake of PFASs can be influenced by other co-contaminants in soil. Plastic film, widely used as greenhouse covers and mulching materials, is a major source of microplastics (MPs, particle size < 5 mm) in plant production systems [5], where MPs are frequently found in conjunction with PFASs through wastewater irrigation, sewage sludge application, and atmospheric deposition [6]. In previous studies, MPs have been identified as vectors for PFASs, altering their bioavailability through direct adsorption mechanisms [7,8] and indirect effects on soil properties as well as plant physiological processes [9]. For instance, Meng et al. [10] investigated the adsorption of perfluorooctanoic acid (PFOA) onto four different types of MPs, with partition coefficients (L/kg) in the order: polystyrene (PS, 2698) > polytetrafluoroethylene (PTFE, 39.6) > polyvinyl chloride (PVC, 23.4) > polyethylene (PE, 17.9). Beyond adsorption, MPs can interfere with plant physiological functions, including photosynthesis, gene expression, oxidative stress responses, and metabolic pathways, thereby influencing PFAS uptake [11].

Although MPs and PFASs widely co-exist in agricultural soils, the effects and mechanisms of MPs on plant accumulation of PFASs remain poorly understood. The effects of MPs on plants vary significantly depending on the types, sizes, shapes, and concentrations. It has been reported that PVC increased plant biomass (54.8%–164.6%), driven by improved soil microbial activity, elevated urease activity, and shifts in microbial community structure [12]. However, Boots et al. [13] provided evidence that biodegradable polylactic acid (PLA) inhibited shoot growth (−19%) and root biomass (−45%) in Lolium perenne by releasing degradation byproducts (e.g., lactic acid), altering soil aggregates, and disrupting chlorophyll-a/b ratios. Tire wear particles (TWP), produced by the mechanical abrasion of tires, were considered a new type of MP and one of the major contributors to MP pollution in the soil environment [14], with a release amount of 5.92 million tons per year [15]. Additionally, it has been reported that TWP inhibited the growth of common beans, though underlying mechanisms remain unclear [16].

In this study, we investigated the effects of different types of MPs on the uptake of PFASs by pak choi (*Brassica chinensis* L.). A total of three types of MPs, namely PVC, PLA, and TWP, with amendment levels of 0.01%, 0.05%, and 0.5% by soil weight, together with 10 PFASs, including 7 legacy and 3 emerging ones, were chosen as representative pollutants. The objectives of this research were to (1) investigate the influence of different MP types on the plant accumulation of PFASs, and (2) explore the mechanisms through multiple pathways, including the adsorption capacities of MPs towards PFASs, the phytotoxicity of MPs, as well as the plant metabolic regulation of PFASs uptake. Elucidating the effects of different MPs on PFASs uptake is essential for assessing combined pollution risks and for informing management strategies for emerging contaminants in agroecosystems.

## 2 Materials and methods

### 2.1 Chemicals and reagents

Three types of MP particles, including PVC, PLA, and TWP, were included in this study. PVC and PLA were purchased from Huachuang Plastic Company (Dongguan, China). TWP was purchased from a commercial tire recycling firm (Nanjing, China). To ensure comparability among treatments, all MPs were standardized prior to use. Specifically, the materials were rinsed with Milli-Q water to remove surface impurities, oven-dried at 40 °C, and sieved to obtain uniform particle sizes. The mean particle sizes of PVC, PLA, and TWP were 110 ± 24 μm, 89.4 ± 24.7 μm, and 53.3 ± 21.4 μm, respectively. The three MPs were characterized using Fourier-transform infrared (FTIR) spectroscopy (NEXUS 870) and scanning electron microscopy (SEM, FEI Nova-450). The FTIR spectra of the three MP types (Fig. S1a–c) were in good agreement with their reference spectra, as reported in previous studies [[17], [18], [19]]. More information about their structural features, specific surface areas, and zeta potential is provided in Fig. S1d–k. The native concentrations of PFASs in these MPs were below the detection limit (0.05−0.54 ng/g).

A total of ten PFASs standards, including four perfluorocarboxylic acids (PFHpA, PFHxA, PFOA, PFNA), three perfluorosulfonic acids (PFBS, PFHxS, PFOS), one replacement compound (HFPO-DA), and two fluorotelomer sulfonic acids (6:2 and 8:2 FTSAs), were obtained from Tokyo Chemical Industry (Japan), Dr. Ehrenstorfer GmbH (Germany), and Columbia Biosciences (U.S.), all with purities above 93%. Detailed information on the standards is provided in Table S1.

Seeds of pak choi (*Brassica chinensis* L.) were purchased from Nanjing Academy of Agricultural Sciences (China). This species was selected due to its widespread consumption in East Asia and its relatively higher PFASs enrichment potential observed in our previous study [20]. The soil sample was collected from Jiangsu Province, China, where the native PFASs concentrations in the soil were below the detection limit (0.05−0.54 ng/g).

### 2.2 Plant exposure experiments

The soil was first spiked with PFASs. Aliquots of 400 g soil were spiked with a PFASs mixture (400 μL stock solution diluted in 10 mL of Milli-Q water) and gradually mixed in multiple increments with continuous stirring for 15 min to ensure homogeneity. Four soil replicates were analyzed after mixing, showing PFASs concentrations ranging from 74.5 ng/g (HFPO-DA) to 148.5 ng/g (8:2 FTSA), with most values near the target (approximately 100 ng/g) and RSDs generally below 10%, confirming homogeneous PFASs distribution (Table S2). The soils were subsequently aged at 20 ± 2 °C for 7 days. Milli-Q water was then added to the soil to achieve a moisture content of 50% of the water-holding capacity. Pot experiments using the PFASs-spiked soil were conducted to evaluate the effects of MP types on the plant uptake of 10 PFASs. Briefly, 3 types of MPs, i.e., PVC, PLA, and TWP, were added to the PFASs-spiked soil at 3 levels: 0.01% (low dose; L), 0.05% (medium dose; M), and 0.5% (high dose; H) of soil weight. The dose settings (0.01%–0.5%) were chosen to cover both realistic and extreme exposure conditions. Concentrations of 0.01% and 0.05% align with the upper levels reported in farmland soils (≤0.054%, Table S3), representing environmentally relevant scenarios. The 0.5% treatment, while above typical farmland values, reflects worst-case conditions occasionally observed in roadside or industrial soils (up to 6.75% and 11.7%) [21], allowing assessment of potential risks under extreme conditions and facilitating the identification of biological responses and mechanistic interactions between MPs and PFASs. This resulted in a total of 11 treatments, including three types of MPs (PVC, PLA, and TWP) at three dose levels (0.01%, 0.05% and 0.5%, w/w in soil), as well as control (PFASs-spiked soil without MPs) and blank (clean soil without either PFASs or MPs) treatments, each with 4 replicates.

Plant uptake experiments were conducted in a greenhouse. Ten germinated pak choi seeds were sown in each pot (10 cm in height, 11 cm in upper diameter, and 10 cm in bottom diameter) containing 400 g of soil. All the pots were randomly placed in a plant growth chamber with the following conditions: day/night (12/12 h) cycle, temperature of 22–26 °C, light intensity of 10,000 lux, and relative humidity of 60%. During the entire plant growth period, pak choi were irrigated using modified Hoagland’s nutrient solutions [20] every 3 days to maintain soil moisture. Forty days after sowing, the plants were harvested and washed three times with ultrapure water, separated into roots and shoots, and then stored at −20 °C for further analysis.

### 2.3 PFAS extraction and instrumental analysis

According to Scordo et al. [22], PFASs extraction from plant tissues was performed using a modified QuEChERS (Quick, Easy, Cheap, Effective, Rugged, and Safe) protocol optimized for this study. Briefly, aliquots of shoot and root samples were treated with acidified acetonitrile and subjected to salting-out extraction, followed by purification on C18, PSA, and GCB sorbents. The extracts were evaporated, reconstituted in water/methanol (1:1, v/v), and filtered prior to liquid chromatography-mass spectrometry (LC-MS/MS) analysis [20]. Detailed extraction and cleanup procedures are provided in Text S1.

PFASs quantification was carried out on a PerkinElmer A30 Altus™ UPLC system coupled to a PerkinElmer Qsight™ 210 triple quadrupole mass spectrometer (LC-MS/MS). Chromatographic separation was performed on a Brownlee SPP C18 column (2.1 mm × 100 mm, 2.7 μm, PerkinElmer, U.S.) maintained at 40 °C, using 5 mmol/L ammonium acetate and methanol as the mobile phases. The detailed analytical parameters can be found in Text S1. Other MS parameters are listed in Table S4. All PFASs concentrations were determined and reported on a dry-weight basis.

### 2.4 Adsorption experiments of MPs towards PFASs

Adsorption experiments were conducted to compare the capacities of different MPs to adsorb PFASs. Each experiment was carried out in a 50 mL polypropylene centrifuge tube containing 200 mg of MPs and 40 mL of PFASs solution, with each congener at a concentration of 1 mg/L. The PFASs concentration (1 mg/L) was selected to differentiate adsorption behaviors among MP types in this high-concentration aqueous experiment, with the caveat that the quantitative results are not intended for direct extrapolation to soil systems. To compare the adsorption capacity between MPs and soil, a control group containing 200 mg of soil without MPs was included. All tubes were shaken at 150 rpm at 25 °C for 4 days, followed by centrifugation at 4000×*g* for 10 min. The resulting supernatants were filtered through 0.22 μm membrane filters and analyzed using UPLC-MS/MS. All experiments were performed in triplicate.

### 2.5 Physiological, biochemical, and molecular characterization in plant

The effects of MP exposure on plants were analyzed by measuring physiological and biochemical indices. Transpiration was quantified on the second or third fully expanded leaf of pak choi using a portable gas-exchange system (LI-6400XT, Li-Cor Inc., U.S.) to record transpiration rate and stomatal conductance. For oxidative stress analysis, approximately 0.1 g of shoot tissue was ground in liquid nitrogen and homogenized in 1 mL of 0.9% saline. The samples were centrifuged at 12,000×*g* and 4 °C for 20 min to remove the residues. The activities of antioxidant enzymes, including superoxide dismutase (SOD), peroxidase (POD), and the content of malondialdehyde (MDA), were quantified using commercial kits (Nanjing Jiancheng Bioengineering Institute, Nanjing, China) with a UV–visible spectrophotometer (UV-1800, Mapada, China).

The hydroponic experiment was designed to explore the potential involvement of aquaporin-mediated pathways in the effects of MPs on PFASs uptake. Briefly, the uniformly sized germinated pak choi seeds were transferred to the hydroponic system containing modified Hoagland’s nutrient solution. Three treatments were prepared with PFASs and MPs at concentrations of 100 ng/L and 0.5%, respectively, to align with the pot and adsorption experiments and ensure a uniform MP dosage. A treatment including PFASs but without MPs was set up in parallel as the control. The 0.5% (w/v) MP dose was applied for mechanistic exploration, with transcriptional responses interpreted qualitatively to reveal plant–MP–PFAS interactions. During exposure, the hydroponic mixtures were manually stirred once daily to maintain a homogeneous MP suspension. After 3 days of exposure, roots and shoots were harvested, rinsed thoroughly with ultrapure water, and stored at −80 °C until analysis.

Total RNA was extracted using the RNeasy Isolation Reagent (Vazyme, Nanjing, China), and first-strand cDNA was synthesized with the HiScript II Synthesis Kit (Vazyme, Nanjing, China). Quantitative real-time PCR was carried out on a LightCycler 96 instrument (Roche, Switzerland) using SYBR Green I fluorescence dye (GenStar, Beijing, China). Four biological and two technical replicates were used in experiments. Primer sequences are listed in Table S5 qPCR data were normalized using EF1α-2, which showed the most stable expression among three candidate reference genes (EF1α-1, EF1α-2, and GAPDH) tested under our conditions. Representative melt-curve profiles of the target genes are shown in Fig. S2. Each primer pair produced a single distinct melting peak, indicating specific amplification without primer-dimer formation. All melt-curve profiles confirmed the specificity and reliability of the qPCR assays.

### 2.6 Metabolomics in plant shoots

To elucidate the metabolic responses of plant shoots to microplastic exposure, untargeted metabolomic profiling was performed using LC-MS/MS (Q Exactive, Thermo Scientific). Each treatment included four biological replicates (n = 4). Data preprocessing, peak alignment, and normalization were conducted in Progenesis QI (Waters Corporation, Milford, U.S.), followed by orthogonal partial least-squares discriminant analysis (OPLS-DA) using the “ropls” package in R. Differential metabolites were identified based on VIP >1 and FDR-adjusted *p* < 0.05. KEGG-based pathway annotation and enrichment analyses were used to interpret the metabolic alterations. Model performance was further evaluated using R^2^X, R^2^Y, and Q^2^ metrics with a 200-time permutation test (Table S6) to confirm model robustness and prevent overfitting. Details on metabolite extraction and instrument conditions are shown in Text S2.

### 2.7 QA/QC and statistical analysis

PFAS concentrations in plants cultivated on non-spiked soils were below the detection limit (0.05−0.54 ng/g). Extraction recoveries, assessed by spiking clean plant matrices with PFAS standards, ranged from 71% to 109%, with detailed congener-specific values provided in Table S7. Mass balance was evaluated as the total PFAS mass retained in plant and soil compartments at the end of exposure relative to the initial spike. The mass balance values ranged from 62.8% ± 6.50% to 79.1% ± 9.68% for different treatments (Fig. S3), suggesting partial PFAS loss due to volatilization, minor leaching, or irreversible sorption to soil constituents. All results were reported as the mean ± standard deviation. One-way ANOVA with Tukey’s HSD post hoc test and paired-sample *t*-tests were performed using SPSS. The statistical significance was set at *p* < 0.05.

## 3 Results and discussion

### 3.1 Plant uptake of PFASs affected by MPs

**Fig. 1:**
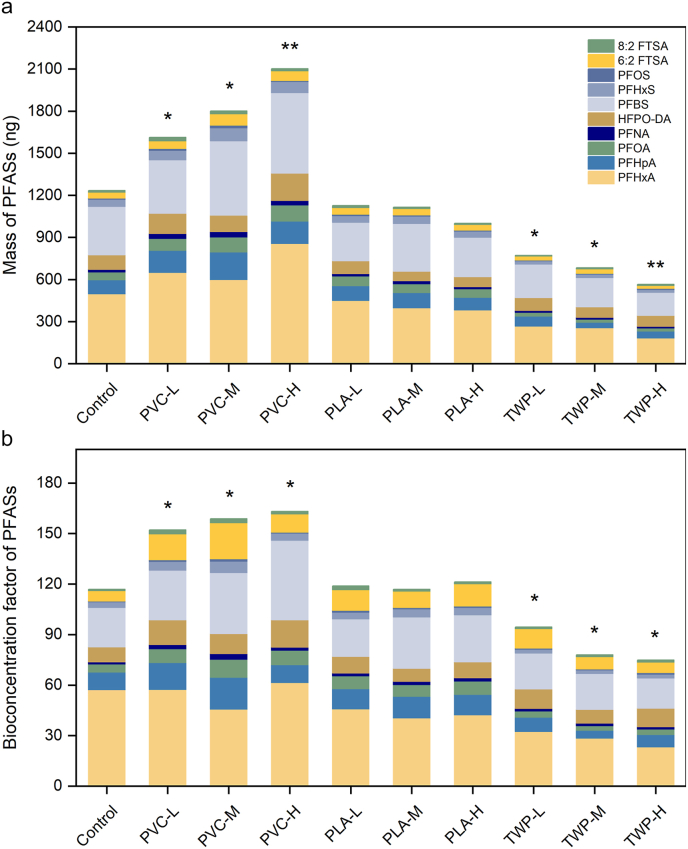
Effect of MP type and dose on PFAS accumulation in shoots. Total mass (a) and bioconcentration factor (b) of 10 PFASs in plant shoots with the amendment of PVC, PLA, and TWP at three levels (L = 0.01%, M = 0.05%, H = 0.5% by soil weight). ∗*p* < 0.05, ∗∗*p* < 0.01.

Total bioaccumulation of 10 PFASs in plants varied markedly depending on MP types (PVC, PLA, and TWP) and doses (0.01%, 0.05%, and 0.5% by soil weight) (Figs. 1 and S4). Given the relatively low levels of PFASs detected in plant roots (Table S8) and the substantial impact of edible parts on human health, our analysis focused on plant shoots. PVC significantly (*p* < 0.05) increased the total mass of 10 PFASs in plant shoots across all the doses (Fig. 1a). Specifically, compared to the control group [1233 ± 122 ng, 95% confidence interval (CI): 1039–1427], PVC amendment significantly (*p* < 0.05) increased PFASs accumulation in shoots by 1.31-, 1.46-, and 1.70-fold, with concentrations of 1613 ± 183 ng (95% CI: 1322–1904), 1801 ± 285 ng (95% CI: 1348–2254), and 2102 ± 504 ng (95% CI: 1300–2904) at 0.01%, 0.05%, and 0.5% amendment levels, respectively. Notably, even at an environmentally relevant level of 0.01%, PVC significantly enhanced PFASs accumulation in the edible parts of vegetables, underscoring the urgent need to consider the interaction between MPs and PFASs for a more comprehensive risk assessment. In contrast, the biodegradable plastic PLA had no significant effect on PFASs uptake in shoots compared with the control, with values of 1127 ± 159 ng (95% CI: 874–1380), 1115 ± 265 ng (95% CI: 693–1537), and 1000 ± 132 ng (95% CI: 790–1210) at doses of 0.01%, 0.05%, and 0.5%, respectively. It is noteworthy that TWP significantly (*p* < 0.05) decreased the uptake of PFASs in shoots across all doses. Specifically, 0.01%, 0.05%, and 0.5% of TWP in soil reduced PFASs mass in shoots by 37.4%, 44.6%, and 54.1%, with concentrations of 772 ± 146 ng (95% CI: 540–1004), 684 ± 106 ng (95% CI: 515–853), and 566 ± 101 ng (95% CI: 405–727), respectively. Consistently, individual PFASs showed similar MP-induced effects as those observed for the total PFASs (Fig. S4). The bioconcentration factor (BCF) of PFASs exhibited a similar trend in Fig. 1b, with the highest values observed under PVC treatment (152–163), followed by PLA (116–121) and the control (117), while TWP exhibited the lowest values (75–95). Zhu et al. [23] reported that the amendment of PS into soil reduced PAHs accumulation in rice by 80.98%, which was attributed to high PAHs adsorption on PS (up to 50%). Conversely, polyethylene terephthalate (PET) exhibited an opposite effect, acting as a vector that facilitated the transfer of PAHs to the rhizosphere zone, thereby promoting the availability of PAHs to the plant [24]. While MP type-dependent behaviors have been noted for other pollutants, our data, for the first time, show a clear MP type-specific modulation of PFASs accumulation in vegetables.

### 3.2 Adsorption of PFASs by MPs and impact on PFAS uptake

**Fig. 2:**
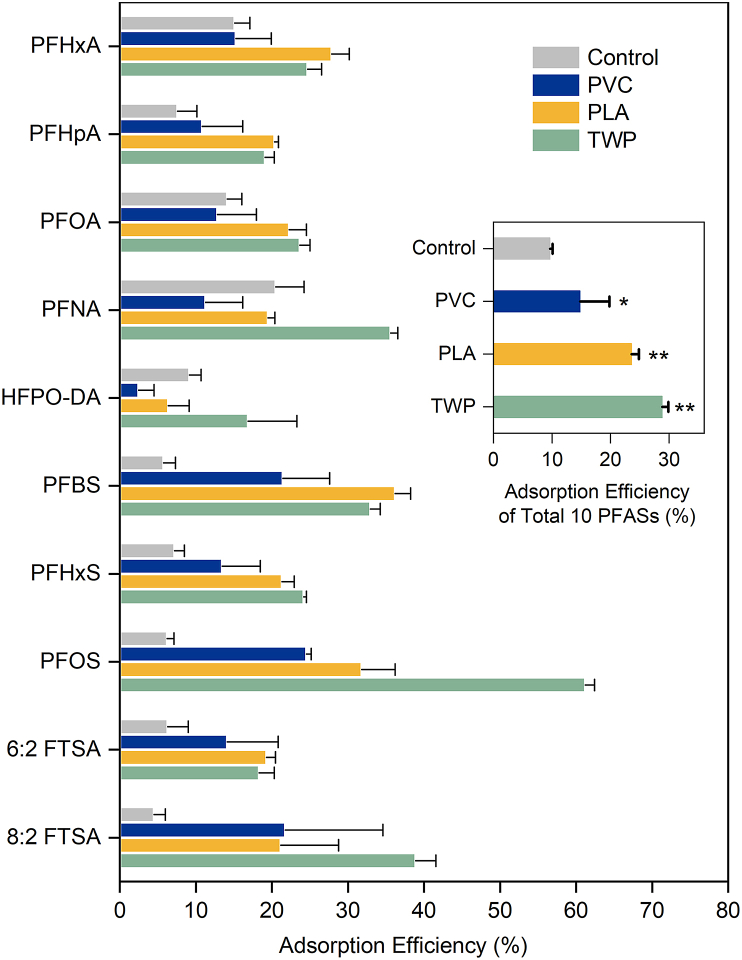
The adsorption efficiency of 10 PFASs by PVC, PLA, and TWP. Soil without MP amendment was used as the control. ∗*p* < 0.05, ∗∗*p* < 0.01.

It has been reported that MPs can serve as vectors for organic pollutants, potentially affecting their bioavailability to plants [8]. In addition, the potential formation of MP–PFAS complexes (e.g., via hydrophobic, electrostatic, or metal-bridging interactions) [25] may further modulate PFASs mobility and plant bioavailability. Therefore, the adsorption of 10 PFASs onto three types of MPs was investigated. The adsorption capacity of each MP was calculated by dividing the amount of adsorbed PFASs by the total amount of PFASs prior to adsorption. Mass-normalized removal efficiencies of MP and aqueous PFASs concentrations after equilibration for each congener are provided in Tables S9 and S10, respectively. As shown in Fig. 2, the total adsorption capacities for the 10 PFASs followed the order: TWP (28.8% ± 1.08%) > PLA (23.6% ± 1.25%) > PVC (14.8% ± 4.99%) > control (9.64% ± 0.45%). Specifically, the adsorption capacities of the 10 individual PFASs ranged from 2.5% (HFPO-DA) to 24.5% (PFOS) for PVC, 6.4% (HFPO-DA) to 36.2% (PFBS) for PLA, and 16.9% (HFPO-DA) to 61.2% (PFOS) for TWP, respectively, compared to the range of 4.48% (8:2 FTSA) to 20.5% (PFNA) for the control. These findings extend prior observations on MP adsorption of other organic contaminants by directly linking MP-specific adsorption to PFASs uptake in plants. For example, Fan et al. [26] found that PLA (1.97 mg/g) had a stronger adsorption capacity for tetracycline compared to PVC (1.36 mg/g). In addition, TWP showed 1.21- and 1.46-fold greater adsorption of chlortetracycline and amoxicillin than PE, respectively, which was attributed to its higher specific surface area and C–O index [14,27]. The relatively stronger adsorption capacity of TWP partially explained the reduction of PFASs uptake observed in TWP-treated plants. In contrast, PFASs uptake was enhanced in PVC treatment, suggesting the involvement of other mechanisms instead of adsorption for PVC.

The differences in adsorption capacity among various MPs can be primarily attributed to their composition and physicochemical properties. MPs can be categorized into glassy and rubbery polymers based on their glass transition temperatures. PVC is classified as a glassy polymer, characterized by its density and extensive cross-linking, which potentially contributes to its relatively low sorption capacity [28]. In contrast, rubbery domains are generally more conducive to the sorption of organic contaminants than glassy domains [28]. TWP, composed primarily of rubber and black carbon [29], exhibited the highest PFASs adsorption capacity among the three MP types. This is likely due to its polar functional groups (e.g., carbonyl and siloxane), rough surface, and smaller particle size (Fig. S1c,f,h), which together provide more available adsorption sites compared to PLA and PVC. In addition, the presence of substantial heavy metals, particularly zinc, as reported by Councell et al. [30] and Rauert et al. [31], may contribute to PFASs sorption via metal-bridging mechanisms. On the other hand, the degradation rate of PLA is inversely related to its crystallinity. To facilitate biodegradability, PLA is often manufactured with low crystallinity, which may impart some rubbery plasticity [32], thereby explaining its stronger adsorption capacity compared to PVC, though still lower than that of TWP.

### 3.3 Phytotoxic effects of MPs on plant growth and PFASs uptake

**Fig. 3:**
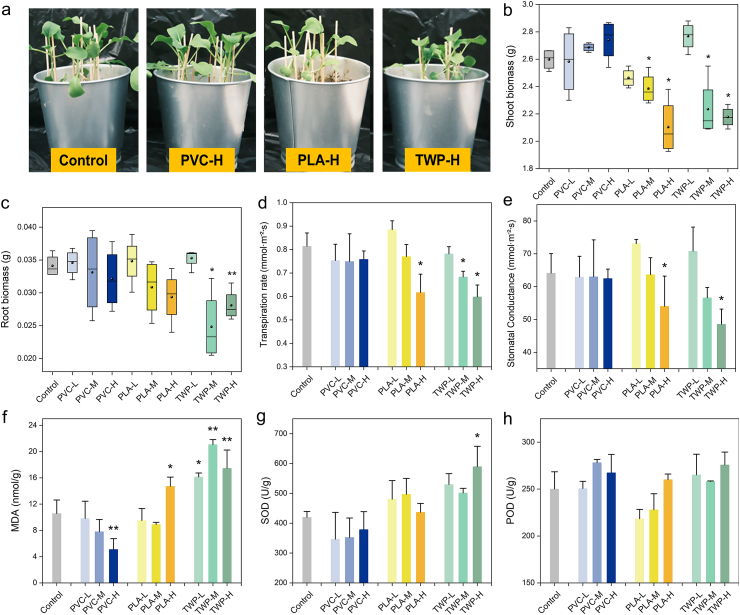
Physiological and biochemical responses of pak choi under MP exposure. Representative images of pak choi seedlings grown in pots treated with PVC, PLA, and TWP at high dose (a). Plant biomass of shoots (b) and roots (c). Transpiration rate (d) and stomatal conductance (e). Malondialdehyde (MDA, f), superoxide dismutase (SOD, g), and peroxidase (POD, h) activities affected by MP exposure at three levels (L = 0.01%, M = 0.05%, H = 0.5% by soil weight). ∗*p* < 0.05, ∗∗*p* < 0.01.

MPs in agricultural soil have been reported to exhibit phytotoxic effects, thus affecting plant growth and, consequently, the accumulation of organic contaminants [33]. To elucidate the differential phytotoxicity among the three MP types, parameters such as plant biomass, transpiration rate, stomatal conductance, oxidative stress markers, and aquaporin gene expression were analyzed. As shown in Fig. 3a–c, PVC did not affect either shoot or root biomass, whereas PLA and TWP reduced plant biomass at both 0.05% and 0.5% doses. Specifically, PLA significantly reduced the shoot biomass by 8.3% (0.05%) and 19.1% (0.5%), while TWP significantly decreased the shoot biomass by 9.2%–16.3% and root biomass by 17.7%–27.3% (*p* < 0.05, Fig. 3b and c). PLA has also been reported to decrease the aboveground height of *Brassica chinensis* L. by 14.2%, likely due to changes in the levels of L-glutamine, L-serine, and arginine, which affect key metabolic pathways governing plant growth and development [18]. Consistent with previous observations on common bean [16], our study demonstrates a comparable inhibitory response in pak choi, highlighting the cross-species relevance of TWP-induced phytotoxicity. TWP, composed of rubber, fillers, oils, vulcanizing agents, and other additives, may exert toxicity through multiple mechanisms, including leachable constituents (e.g., Zn^2+^ was measured at 7.8 ± 1.0 mg/kg in TWP in this study), particle-induced stress, and alterations of soil physicochemical properties [15,16].

Transpiration is another important physiological parameter closely related to the translocation of xenobiotic chemicals from root to shoot, especially for ionic and water-soluble organic pollutants like PFASs [34]. Transpiration rate and stomatal conductance are key indicators of water transport efficiency in plants. As shown in Fig. 3d and e, at the 0.5% dose, PLA reduced the transpiration rate and stomatal conductance to 76% and 84% of the control, respectively, while TWP reduced them to 73% and 76% of the control; in contrast, PVC showed no significant effect. Yu et al. [35] reported that reduced transpiration rate and stomatal conductance led to decreased PFAS accumulation in plant shoots. Accordingly, a positive relationship was observed in the TWP group, where a 27% reduction in transpiration rate coincided with a 54.1% decrease in shoot PFASs accumulation at the 0.5% dose, suggesting that transpiration likely contributes to PFASs transport within the plant. These results suggest that TWP reduced PFASs bioaccumulation primarily via its inhibitory effects on plant growth and transpiration. In contrast, although PLA also suppressed biomass and transpiration, its lack of effect on PFASs accumulation indicated the involvement of other compensatory mechanisms.

Oxidative stress, triggered by reactive oxygen species (ROS), is a key mechanism underlying MP-induced phytotoxicity [5]. Biochemical indicators, such as MDA levels and antioxidant enzyme activities (e.g., SOD and POD), are commonly used to evaluate oxidative damage in plants. MDA, an indicator of oxidative membrane damage, can reflect alterations in intrinsic cell membrane characteristics such as fluidity and ion transport [36]. In our study, MDA content was significantly (*p* < 0.05) increased by TWP across all doses, while PLA only caused a significant increase at the high dose (0.5%), further confirming the stronger phytotoxicity of TWP (Fig. 3f–h). MPs have been reported to induce oxidative stress and interfere with the uptake of other organic pollutants, whereas studies on their co-exposure with PFASs remain very limited. For example, a 28-day hydroponic study found that PE increased SOD, CAT, and MDA in both roots and shoots of lettuce exposed to di-n-butyl phthalate (DBP) [37], potentially reducing the uptake of DBP due to oxidative damage to plant cell structure and function [38]. Another study by Xu et al. [39] demonstrated that PS reduced the uptake of phenanthrene in soybean roots and leaves by inducing genotoxic and oxidative damage. These findings suggest that TWP-induced oxidative stress may partially explain the reduced PFASs uptake observed in plants by impairing membrane-mediated transport.

**Fig. 4:**
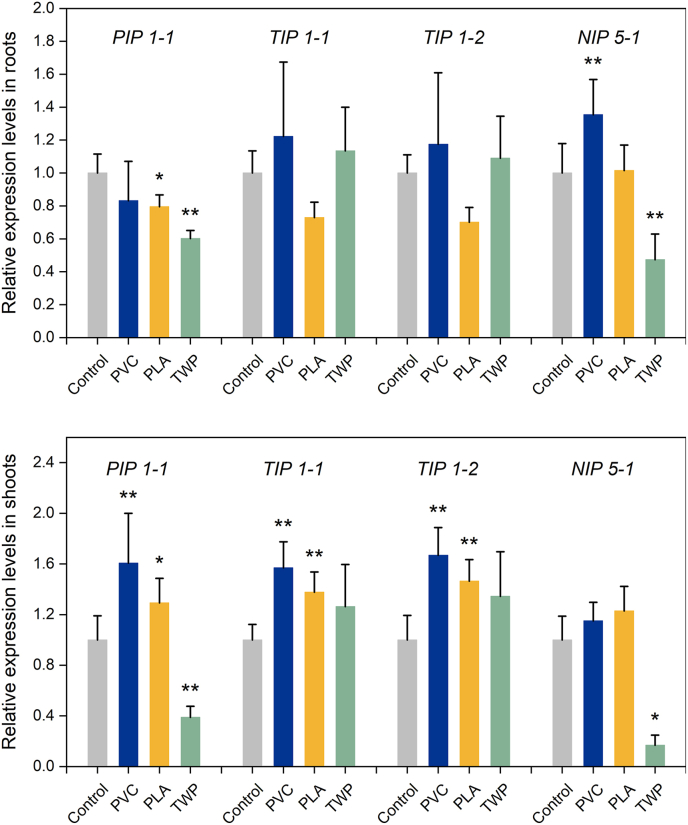
Relative expression levels of aquaporin genes in roots and shoots with the amendment of PVC, PLA, and TWP at a dose of 0.5% compared to the control. ∗*p* < 0.05, ∗∗*p* < 0.01.

Previous studies using inhibitor assays have demonstrated that PFASs uptake can be mediated by aquaporins [20,40], providing experimental evidence for the involvement of aquaporin pathways in PFASs translocation. To further investigate the molecular mechanisms underlying MP effects on the plant uptake of PFASs, the expression of aquaporin genes was determined. Four aquaporin genes, i.e., *PIP1-1, TIP1-1*, *TIP1-2*, and *NIP5-1*, encoding aquaporins localized to different cellular membranes, play important roles in osmoregulation and nutrient transport [41]. As shown in Fig. 4, MP treatments induced differential expression of these aquaporin genes. Compared with the control, PVC exposure significantly (*p* < 0.05) upregulated the expression of *PIP1-1*, *TIP1-1*, and *TIP1-2* in plant shoots by 1.57- to 1.67-fold, as well as *NIP5-1* by 1.35-fold in roots, suggesting enhanced water-transport capacity that could facilitate PFASs accumulation. Similarly, previous studies have shown that PS exposure upregulated the expression of aquaporin genes such as *TIP2-1, TIP2-2, PIP2-6, PIP2-8, SIP2-1,* and *NIP1-2* by 1.5- to 3.0-fold; notably, PS and PVC are both glassy polymers [36]. In contrast, TWP exposure significantly (*p* < 0.05) downregulated the expression of *NIP5-1* and *PIP1-1* by 0.47- to 0.60-fold in roots and 0.17- to 0.39-fold in shoots, which was consistent with its reduction effect on plant transpiration. It has been reported that aquaporins are closely correlated with transpiration in plants [42], suggesting that the reduced PFASs uptake under TWP exposure may be linked to the impaired water-transport capacity. The mechanistic effects of PLA appeared contradictory. On one hand, it induced phytotoxic effects, such as biomass reduction and oxidative stress, which may reduce the PFASs uptake. On the other hand, it promoted the expression of aquaporin genes, such as *PIP1-1*, *TIP1-1*, and *TIP1-2* in plant shoots by 1.29- to 1.46-fold, suggesting a possible role of aquaporin-associated water transport in facilitating PFASs movement within plants. These opposing effects may potentially offset each other and thus could help explain the limited overall impact of PLA on plant PFASs uptake. Organic matter is a key factor governing PFASs sorption [43]. In our study, amendment with PVC-H, PLA-H, and TWP-H increased soil organic matter by 4%, 39%, and 46%, respectively (Table S11), which likely enhanced PFASs adsorption in PLA- and TWP-amended soils compared to PVC. TWP also increased soil fluorescein diacetate hydrolytic activity by 46%, indicating pronounced changes in soil enzyme activity and supporting its toxic effects. By contrast, soil pH and CEC showed little variation among treatments.

Overall, PFASs uptake was governed by distinct processes among MP types. In brief, PVC enhanced the uptake primarily through physiological regulation, TWP reduced the PFASs accumulation through its strong sorption affinity for PFASs together with toxicity-induced inhibition, and PLA exerted only a limited influence due to moderate sorption capacity and counteracting biological effects. These findings highlight the varying dominance of adsorption and plant physiological responses across MP types. Nonetheless, differences in particle size and surface area may also contribute to the observed effects, suggesting that future studies should control these variables to better isolate material-specific mechanisms.

### 3.4 Metabolic responses to MP exposure and PFASs accumulation

**Fig. 5:**
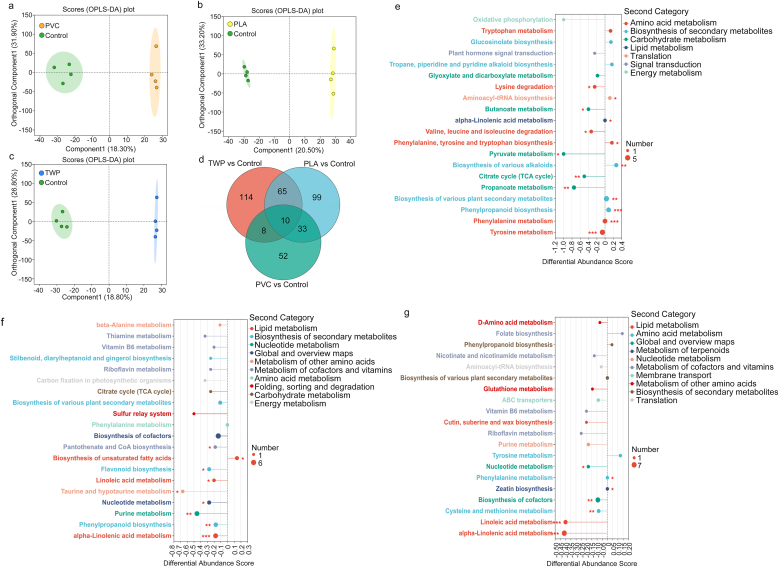
Metabolic profiling and pathway enrichment analyses of plant shoots in response to MP exposure at a 0.5% dose. Orthogonal partial least-squares discriminant analysis (OPLS-DA) of shoot metabolites under PVC (a), PLA (b), TWP (c); Venn diagram between control and PVC, PLA, TWP (d); Differential abundance scores of KEGG pathways of plant shoots with the amendment of PVC (e), PLA (f), TWP (g).

To reveal the metabolic responses of plant shoots under exposure to different types of MPs, untargeted metabolomic profiling was conducted, and OPLS-DA was performed at a 0.5% dose, as this dose showed the most pronounced impact on PFAS accumulation. The OPLS-DA loading plots revealed distinct separations between the control and each MP treatment group, namely PVC, PLA, and TWP, on the first principal component, accounting for 18.3%, 20.5%, and 18.8% of the total variance, respectively (Fig. 5a–c). These results demonstrated that the amendment of MPs induced distinct MP type-specific alterations in plant shoot metabolism. According to the Venn diagram (Fig. 5d), there were 52, 99, and 114 specific differential metabolites in PVC-, PLA-, and TWP-treated groups compared with the control, indicating that TWP exerted the most extensive metabolic disruption among the three MPs. Based on VIP >1, the numbers of significantly altered metabolites included 31, 38, and 21 upregulated, and 72, 169, and 176 downregulated metabolites for PVC, PLA, and TWP, respectively (Fig. S5). These metabolites primarily belonged to lipids, amino acids and their derivatives, as well as carbohydrates and their derivatives (Fig. S6).

KEGG pathway enrichment analysis was conducted to identify specific metabolic pathways affected by MP exposure (Tables S12-S14). Detailed lists of the top 30 differential metabolites (VIP >1, *p* < 0.05) identified under PVC-H, PLA-H, and TWP-H treatments are provided in Tables S15-S17. Overall, PVC exposure induced moderate metabolic responses distinct from those caused by PLA and TWP (Figs. 5e and S7). In contrast, PLA and especially TWP exposures predominantly downregulated key metabolic pathways, particularly those related to lipid and amino acid metabolism (Fig. 5f and g). Notably, the downregulatory effects of TWP on lipid metabolism were more pronounced than those of PLA, as evidenced by more negative differential abundance (DA) scores (e.g., −0.41 for TWP vs. −0.18 for PLA in α-linolenic acid metabolism). The inhibition of lipid metabolism pathways was accompanied by a significant reduction in unsaturated fatty acid derivatives in plant shoots. For instance, arachidonic acid was significantly downregulated in the PLA-treated group (Table S16), while 2,13-trans-Epoxy-9-oxo-10E,15Z-octadecadienoic acid (an oxylipin intermediate of linoleic acid metabolism and a functional analog of dn-OPDA) was suppressed under TWP exposure (Table S17) [44]. These metabolites are involved in oxylipin biosynthesis and membrane lipid remodeling, suggesting that both PLA and TWP may disrupt lipid-mediated stress signaling and membrane integrity [45]. This was consistent with the increased MDA levels and the reduced PFASs accumulation in plant tissues, particularly under TWP exposure (Fig. 3f). Similarly, Hanano et al. [46] demonstrated that the changes in lipid metabolism pathways were directly related to the accumulation of tetrachlorodibenzo-p-dioxin in Arabidopsis. Additionally, amino acid metabolism was significantly (*p* < 0.05) downregulated in both PLA and TWP groups, for example, taurine and hypotaurine in PLA (DA score = −0.67), as well as cysteine and methionine in TWP (DA score = −0.08) (Fig. 5f and g). Since amino acids are primary nitrogen reserves and play crucial roles in plant development and defense [47], their reduction could partly explain the observed growth inhibition. Notably, TWP also down-regulated the content of 12-oxo-2,3-dinor-10,15-phytodienoic acid in plant shoots (Table S17), which is a derivative of the jasmonic acid pathway [48] that regulates stomatal closure and transpiration [49]. This result aligned with our physiological findings that TWP reduced transpiration rate and stomatal conductance, ultimately leading to decreased PFAS uptake in shoots. Metabolic alterations under MP exposure, especially in lipid and phytohormone pathways, are consistent with the observed reductions in transpiration and PFAS uptake, providing a plausible though inferential mechanistic explanation.

## 4 Conclusions

This study investigated the impact of MP types on PFASs uptake by vegetables (pak choi) at levels of 0.01%, 0.05%, and 0.5% MPs in soil. Even with an amendment of PVC at only 0.01%—a level comparable to concentrations reported in farmland soils [50]—a significant increase in PFASs uptake by pak choi was observed. This implied that environmental levels (e.g., 0.01%) of MPs may contribute to enhanced human exposure risk to PFASs through food consumption. Conversely, the reduction in PFASs uptake associated with TWP was attributed to its phytotoxicity, which raised another concern about its ecological impacts, such as reduced crop production. Overall, our study advances the understanding of MP–PFAS co-contamination by combining systematic comparison with multi-omics mechanistic insights. The findings reveal that the effects of MPs on PFASs uptake are strongly type-specific, emphasizing the critical role of polymer characteristics in governing contaminant behavior. To prevent MPs and PFASs from entering the food chain, management bodies should strengthen oversight of biodegradable plastic mulches (like PLA) and prioritize the monitoring of roadside and industrial soils, which often have higher contamination levels. Future research should adopt a comprehensive approach, considering factors such as the type, dose, and phytotoxicity of MPs, as well as multiple crop species, to develop a more nuanced understanding of their impact on both the health and ecological risks of contaminants.

## CRediT authorship contribution statement

**Qian Gu:** Writing – review & editing, Writing – original draft, Methodology, Investigation, Conceptualization. **Pengfei Zhou:** Validation, Methodology, Investigation. **Yi Kong:** Validation, Software, Methodology. **Chenzhuo Song:** Software, Methodology, Investigation, Data curation. **Qian Zhang:** Writing – review & editing, Writing – original draft, Supervision, Funding acquisition, Conceptualization. **Xinyi Cui:** Writing – review & editing, Validation, Supervision, Methodology, Investigation, Conceptualization.

## Data availability

The metabolomics data supporting this study are publicly available at https://doi.org/10.5281/zenodo.17543827. Other data supporting the findings of this work are available within the article and its Supplementary Information, or from the corresponding author upon reasonable request.

## Declaration of competing interest

The authors declare no competing financial interest or personal relationships that could have appeared to influence the work reported in this paper.
