# Supplementary material for: Effects and mechanisms of microplastic types on plant uptake of per- and polyfluorinated alkyl substances
Source: Eco Environ Health. 2026 Jan 19;5(1):100216. doi: 10.1016/j.eehl.2026.100216 (PMC12907065; doi:10.1016/j.eehl.2026.100216)
Supplement: Multimedia component 1 [file mmc1.docx]

**Supporting Information**

**Effects and Mechanisms of Microplastic Types on** **Plant Uptake of** **Per- and Polyfluorinated Alkyl Substances**

Qian Gu, Pengfei Zhou, Yi Kong, Chenzhuo Song, Qian Zhang^*^, Xinyi Cui^*^

State Key Laboratory of Water Pollution Control and Green Resource Recycling, School of the Environment, Nanjing University, Nanjing 210023, China

**Corresponding authors**

E-mail: [qianzhang326@nju.edu.cn](mailto:qianzhang326@nju.edu.cn) (Q. Zhang); [lizzycui@nju.edu.cn](mailto:lizzycui@nju.edu.cn) (X. Cui)

Number of pages: 36

Number of texts: 3

Number of tables: 17

Number of Fig.s: 7

**Table of Contents**

Text S1. PFAS Extraction and Instrumental analysis.

Text S2. Metabolomics in plant shoots.

Text S3. Determination of Soil Properties and Zn Concentration in TWP.

Table S1. Detailed information about 10 PFASs.

Table S2. Spiked concentrations of 10 PFASs in soil samples (ng/g).

Table S3. Soil microplastic concentrations reported in the literature.

Table S4. LC-MS/MS instrument parameters for quantification of the target analytes.

Table S5. Primers used for quantitative real-time PCR.

Table S6. OPLS-DA model performance metrics for metabolic differentiation among treatments

Table S7. Congener-specific recoveries of 10 PFASs.

Table S8. Mass of individual PFAS in plant roots with the amendment of PVC, PLA, and TWP (ng).

Table S9: Mass-normalized PFAS removal (μg PFAS/g MP) for each congener

Table S10: Aqueous PFAS concentrations after equilibration for each congener in the adsorption experiment.

Table S11. Soil physical and chemical properties under MP exposure at 0.5% dose.

Table S12. KEGG pathway enrichment analysis for the PVC-H treatment.

Table S13. KEGG pathway enrichment analysis for the PLA-H treatment.

Table S14. KEGG pathway enrichment analysis for the TWP-H treatment.

Table S15. Top 30 metabolites with VIP > 1 in plants shoots exposed to PVC-H.

Table S16. Top 30 metabolites with VIP > 1 in plants shoots exposed to PLA-H.

Table S17. Top 30 metabolites with VIP > 1 in plants shoots exposed to TWP-H.

Fig. S1. The structural and morphological characterization of MPs.

Fig. S2. Representative melt-curve profiles for qPCR amplifications of target genes: (a) *PIP1-1*, (b) *TIP1-1*, (c) *TIP1-2*, and (d) *NIP5-1.*

Fig. S3. Mass balance of total 10 PFASs in the whole plant exposure experiments with the treatment of MPs.

Fig. S4. Bioconcentration of individual PFAS in plant shoots with the amendment of PVC, PLA, and TWP.

Fig. S5. Volcano maps showing differential metabolites in plant shoots between control and PVC, PLA, and TWP.

Fig. S6. Primary metabolites in plant shoots with the amendment of PVC, PLA, and TWP.

Fig. S7. Differential abundance score of KEGG pathways of plant shoots with the amendment of PVC, PLA, and TWP.

Text S1. PFAS Extraction and Instrumental analysis.

PFASs were extracted from freeze-dried plant tissues using a modified QuEChERS method [1]. In brief, plant samples were freeze-dried for 48 h, and then aliquots of 0.05 g of root and 0.1 g of shoot were added to 15 mL centrifuge tubes, respectively. Subsequently, 1 mL ultrapure water and 2 mL hydrochloric acid/acetonitrile mixture (0.2:99.8, v/v) were added to the tubes. The tubes were shaken manually for 15 s, vortex-mixed for 10 min, and sonicated for 30 min. Aliquots of 0.5 g sodium chloride was added, and the mixture was vortex-mixed for another 10 min. The tubes were centrifuged at 4000×*g* for 10 min. The acetonitrile supernatant was transferred to 15 mL centrifuge tubes containing 100 mg GCB, 80 mg C18, and 100 mg PSA for purification. The tubes were then vortex-mixed for 10 min, and centrifuged at 4000×*g* for 5 min. Subsequently, 1 mL acetonitrile supernatant was transferred to 5 mL centrifuge tube, dried under a gentle stream of nitrogen, redissolved in 0.25 mL ultrapure water/methanol (1:1, v/v), filtered through 0.22 μm polypropylene membrane, and stored at -20 °C until analysis.

The analysis of PFASs was performed on a PerkinElmer A30 Altus™ UPLC system with a PerkinElmer Qsight™ 210 triple quadrupole mass spectrometer. The experimental conditions for ESI-MS/MS were as following: Drying gas: 150 psi, HSID temperature: 250 ℃, Nebulizer Gas: 300 psi, Electrospray V1 Neg: -4500 V, and Source 1 temperature: 200 ℃. The detailed MS transition and parameters for each analyte are shown in Table S3. Analytes were separated using a brownlee SPP C18 column (2.1 mm × 100 mm, 2.7 μm, PerkinElmer, U.S.) at 40 ℃, while 5 mmol/L ammonium acetate (A) and 100% methanol (B) were used as the mobile phase, running in the gradient mode. The initial gradient (10% B) was held for 1 min, then ramped to 30% B at 2 min and then changed to 30 (A):70 (B) for further 7 min, then 85% B for 2 min and 90% B for 1 min, and finally changed to 90 (A):10 (B) with a post-run (wash) for 2 min. The flow rate was at 0.3 ml/min. Total analysis time was 15 min per sample for 10 PFASs.

Text S2. Metabolomics in plant shoots

Briefly, 100 mg shoots were added to a 2 mL centrifuge tube and a 6 mm diameter grinding bead was added. Then, 400 μL of extraction solution (methanol : water = 4:1 (v:v)) containing 0.02 mg/mL of internal standard (L-2-chlorophenylalanine) was used for metabolite extraction. Samples were ground by the Wonbio-96c (Shanghai wanbo biotechnology co., LTD) frozen tissue grinder for 6min (-10 °C, 50 Hz), followed by low-temperature ultrasonic extraction for 30 min (5 °C, 40 kHz). The samples were left at-20 °C for 30 min, centrifuged for 15 min (4 °C, 13000×*g*), and the supernatant of shoots were transferred to the injection vial for LC-MS/MS analysis.

The LC-MS/MS analysis of shoots was conducted on a Thermo UHPLC-Q Exactive system equipped with an ACQUITYHSS T3 column (100 mm × 2.1 mm × 1.8 μm; Waters, U.S.). The mobile phases consisted of 0.1% formic acid in water: acetonitrile (98:2, v/v) (solvent A) and 0.1% formic acid in acetonitrile (solvent B). The flow rate was 0.40 mL/min and the column temperature was 40 ℃. The UPLC system was coupled to a Thermo UHPLC-Q Exactive Mass Spectrometer equipped with an electrospray ionization (ESI) source operating in positive mode and negative mode. The optimal conditions were set as followed: source temperature at 400 ℃; sheath gas flow rate at 40 arb; Aux gas flow rate at 10 arb; ion-spray voltage floating (ISVF) at-2800V in negative mode and 3500V in positive mode, respectively; Normalized collision energy, 20-40-60V rolling for MS/MS. Full MS resolution was 70000, and MS/MS resolution was 17500. Data acquisition was performed with the Data Dependent Acquisition (DDA) mode. The detection was carried out over a mass range of 70−1050 *m/z*.

The pretreatment of raw data was performed by Progenesis QI (Waters Corporation, Milford, U.S.) software. The R package “ropls” (Version 1.6.2) was used to perform orthogonal partial least-squares discriminant analysis (OPLS-DA). Differential metabolites were screened by T. test and variable importance in projection (VIP). Differential metabolites among two groups were mapped into their biochemical pathways through metabolic enrichment and pathway analysis based on KEGG database (http://www. genome.jp/kegg/). These metabolites could be classified according to the pathways they involved or the functions they performed. Enrichment analysis was used to analyze a group of metabolites in a function node whether appears or not. The principle was that the annotation analysis of a single metabolite develops into an annotation analysis of a group of metabolites. Python packages “scipy.stats” (https://docs.scipy.org/doc/scipy/) was used to perform enrichment analysis to obtain the most relevant biological pathways for experimental treatments.

Text S3. Determination of Soil Properties and Zn Concentration in TWP.

The effects of MPs on soil chemical properties were analyzed after plant harvest. Soil pH was measured potentiometrically at a soil-to-water ratio of 1:2.5 (w/v). Total organic carbon (TOC) was determined using a TOC analyzer (Shimadzu, Japan). Cation exchange capacity (CEC) was measured by ammonium acetate extraction (1 mol/L, pH 7.0), in which soil samples were first saturated with ammonium ions and subsequently displaced with sodium to quantify exchangeable cations. Soil fluorescein diacetate hydrolytic activity (S-FDA), representing overall microbial enzyme activity, was quantified using commercial assay kits (Nanjing Jiancheng Bioengineering Institute, China) and a UV–visible spectrophotometer (UV-1800, Mapada, China).

For Zn analysis, 0.05 g of tire wear particles (TWP) were mixed with 10 mL of Milli-Q water (liquid-to-solid ratio = 200 L/kg and pH = 7.0), shaken at 25 °C and 100 rpm for 2 h, and centrifuged at 4000×*g* for 10 min. The supernatant was then filtered through 0.45 µm membrane filters prior to ICP-OES measurement.

Table S1. Detailed information about 10 PFASs.

| Category | Abbreviation | Full name | Molecular Formula | CAS Number |
| --- | --- | --- | --- | --- |
| PFCA | PFHxA | Perfluorohexanoic acid | CF_3_(CF_2_)_4_COOH | 307-24-4 |
|  | PFHpA | Perfluoroheptanoic acid | CF_3_(CF_2_)_5_COOH | 375-85-9 |
|  | PFOA | Perfluorooctanoate acid | CF_3_(CF_2_)_6_COOH | 335-67-1 |
|  | PFNA | Perfluorononanoic acid | CF_3_(CF_2_)_7_COOH | 375-95-1 |
|  | HFPO-DA | Hexafluoropropylene oxide dimer acids | CF_3_(CF_2_)_2_O(CF_2_)_2_COOH | 13252-13-6 |
| PFSA | PFBS | Perfluorobutanesulfonic acid | CF_3_(CF_2_)_3_SO_3_H | 375-73-5 |
|  | PFHxS | Perfluorohexanesulfonic acid | CF_3_(CF_2_)_5_SO_3_H | 355-46-4 |
|  | PFOS | Perfluorooctanesulfonate | CF_3_(CF_2_)_7_SO_3_H | 1763-23-1 |
|  | 6:2 FTSA | 6:2 Fluorotelomer sulfonic acid | CF_3_(CF_2_)_5_(CH_2_)_2_SO_3_H | 27619-97-2 |
|  | 8:2 FTSA | 8:2 Fluorotelomer sulfonic acid | CF_3_(CF_2_)_7_(CH_2_)_2_SO_3_H | 39108-34-4 |

Table S2. Spiked concentrations of 10 PFASs in soil samples (ng/g).

|  |  | PFHxA | PFHpA | PFOA | PFNA | HFPO-DA | PFBS | PFHxS | PFOS | 6:2 FTSA | 8:2 FTSA |
| --- | --- | --- | --- | --- | --- | --- | --- | --- | --- | --- | --- |
| Control | Average | 127.5 | 123.8 | 112.8 | 129.8 | 78.9 | 101.2 | 100.5 | 91.3 | 104.6 | 101.1 |
|  | RSD (%) | 6.8 | 5.2 | 3.5 | 3.0 | 6.8 | 6.6 | 3.7 | 9.3 | 5.1 | 8.5 |
| PVC-L | Average | 114.6 | 115.9 | 104.9 | 113.9 | 74.5 | 95.2 | 97.3 | 91.8 | 99.7 | 86.0 |
|  | RSD (%) | 4.6 | 3.5 | 6.8 | 10.1 | 9.8 | 2.4 | 2.9 | 6.0 | 7.3 | 9.3 |
| PVC-M | Average | 105.7 | 118.3 | 107.2 | 109.2 | 75.0 | 96.4 | 104.7 | 101.6 | 101.7 | 96.5 |
|  | RSD (%) | 4.7 | 2.6 | 5.9 | 7.6 | 6.0 | 5.6 | 2.0 | 3.1 | 5.5 | 14.0 |
| PVC-H | Average | 108.8 | 117.2 | 109.0 | 117.8 | 74.9 | 95.5 | 103.5 | 101.1 | 105.7 | 90.2 |
|  | RSD (%) | 6.6 | 5.2 | 10.6 | 8.3 | 3.9 | 4.5 | 5.7 | 9.7 | 9.9 | 7.7 |
| PLA-L | Average | 116.4 | 130.0 | 120.1 | 126.0 | 84.4 | 105.1 | 114.1 | 118.6 | 110.3 | 94.0 |
|  | RSD (%) | 4.0 | 3.9 | 8.0 | 1.4 | 3.1 | 2.0 | 3.4 | 5.6 | 3.6 | 13.2 |
| PLA-M | Average | 109.3 | 121.9 | 115.0 | 120.2 | 78.3 | 96.0 | 100.7 | 100.8 | 98.3 | 96.2 |
|  | RSD (%) | 5.1 | 4.7 | 5.7 | 5.8 | 12.4 | 4.2 | 4.1 | 10.0 | 6.0 | 14.9 |
| PLA-H | Average | 126.1 | 134.1 | 127.7 | 126.7 | 90.5 | 95.1 | 97.9 | 87.0 | 105.5 | 103.7 |
|  | RSD (%) | 7.3 | 9.1 | 9.0 | 11.7 | 8.8 | 8.5 | 9.5 | 16.8 | 5.4 | 0.6 |
| TWP-L | Average | 112.7 | 120.5 | 118.9 | 126.9 | 75.4 | 93.2 | 95.6 | 100.1 | 99.1 | 112.7 |
|  | RSD (%) | 3.1 | 3.4 | 8.3 | 6.3 | 8.4 | 2.8 | 4.1 | 14.1 | 7.6 | 11.7 |
| TWP-M | Average | 132.9 | 138.9 | 138.8 | 141.3 | 83.6 | 96.2 | 99.9 | 101.0 | 106.5 | 148.5 |
|  | RSD (%) | 7.2 | 6.0 | 3.3 | 1.9 | 8.8 | 8.4 | 6.1 | 9.0 | 5.4 | 2.7 |
| TWP-H | Average | 110.9 | 119.1 | 100.5 | 109.4 | 80.2 | 100.3 | 110.8 | 98.5 | 108.3 | 108.3 |
|  | RSD (%) | 5.0 | 5.1 | 2.5 | 1.0 | 7.0 | 3.5 | 4.3 | 6.1 | 3.9 | 4.2 |

Table S3. Soil microplastic concentrations reported in the literature.

| **Location** | **Field type** | **Major source of microplastics** | **Microplastic concentration** | | **Reference** |
| --- | --- | --- | --- | --- | --- |
|  |  |  | **Mass concentration** | **% w/w** |  |
| Germany | Potting soil | - | 0.03-3.3 mg/g | 0.003-0.33 | [2] |
| China | Farmland | Plastic mulching | 9.25-369.55 mg/kg | 0.001- 0.04 | [3] |
| China | Farmland | Plastic mulching | average: 368 mg/kg | 0.037 | [4] |
| China | Fruit field | Plastic mulching | average: 540 mg/kg | 0.054 | [4] |
| France | Farmland | Compost | average: 417.4 kg/ha | 0.016 | [5] |
| Denmark | Farmland | - | 0.224 g/kg | 0.022 | [6] |
| Chile | Farmland | Biosolids | 0.57-12.9 mg/kg | <0.002 | [7] |
| Canada | Farmland | Biosolids | 2.3-28.5 mg/kg | <0.003 | [8] |
| Sydney | Industrial area | Historical plastic production | 300-67,500 mg/kg | 0.030-6.75 | [9] |
| France, Japan, U.S. | Roadside soil | Tire wear particle | 20 g/kg | 2 | [10] |
| Global | Roadside soil | Tire wear particle | 2.3 to 117 g/kg | 0.023-11.7 | [11] |

Table S4. LC-MS/MS instrument parameters for quantification of the target analytes.

| Analytes | Precursor ion | Product ion | Retention time (min) | EV (V) | CCL2 (V) | CE (V) |
| --- | --- | --- | --- | --- | --- | --- |
| PFHxA | 313→269 | 313→119 | 6.89 | -1 | 72 | 13 |
| PFHpA | 363→319 | 363→169 | 8.17 | -3 | 101 | 13 |
| PFOA | 413→369 | 413→169 | 9.16 | -5 | 104 | 13 |
| PFNA | 463→419 | 463→219 | 9.99 | -5 | 134 | 16 |
| HFPO-DA | 495→186 | 495→119 | 3.47 | -3 | 56 | 28 |
| PFBS | 300→80 | - | 5.68 | -40 | 89 | 50 |
| PFHxS | 400→80 | 400→99 | 8.29 | -30 | 133 | 54 |
| PFOS | 500→80 | 500→99 | 10.02 | -33 | 204 | 66 |
| 6:2 FTSA | 427→407 | 427→81 | 9.11 | -10 | 144 | 35 |
| 8:2 FTSA | 527→507 | 527→81 | 10.68 | -39 | 128 | 36 |

EV: Entrance Voltage; CCL2: Collision Cell Lens 2; CE: Collision Energy

Table S5. Primers used for quantitative real-time PCR.

| Gene name | Primer sequence (5'-3') |
| --- | --- |
| Actin-F | ATACCAGGCTTGAGCATACCG |
| Actin-R | GCCAAAGAGGCCATCAGACAA |
| PIP1-1-F | GCTGCACTTGCTGCTCTTTA |
| PIP1-1-R | AGGACACGGGATCAGAAACC |
| TIP1-1-F | ATTGGCAGGTCCTCAGTTGG |
| TIP1-1-R | TGGTTCACCTCTGTGGCATC |
| TIP1-2-F | TCCGTGGTCTCCTCTACTGG |
| TIP1-2-R | GAACGCTGGAACTGCCAAG |
| NIP5-1-F | CGACCTCGTCTCGTCATTGT |
| NIP5-1-R | AAACAGCCTTGTCACCGAGG |

Table S6. OPLS-DA model performance metrics for metabolic differentiation among treatments

|  | R²X | R²Y | Q² | Permutation test (*p*) | Model validity |
| --- | --- | --- | --- | --- | --- |
| PVC vs Control | 0.502 | 0.989 | 0.652 | < 0.05 (6/200) | Valid |
| PLA vs Control | 0.693 | 0.999 | 0.824 | < 0.05 (0/200) | Valid |
| TWP vs Control | 0.681 | 0.997 | 0.739 | < 0.05 (5/200) | Valid |

Notes: R²X and R²Y represent the explained variance of the X and Y matrices, respectively, and Q² indicates the predictive ability of the model. Permutation tests (200 iterations) confirmed that all models were robust without overfitting (*p* < 0.05).

Table S7. Congener-specific recoveries of 10 PFASs

|  | PFHxA | PFHpA | PFOA | PFNA | HPFO-DA | PFBS | PFHxS | PFOS | 6:2 FTSA | 8:2 FTSA |
| --- | --- | --- | --- | --- | --- | --- | --- | --- | --- | --- |
| AVE (%) | 92.6 | 96.4 | 83.4 | 91.9 | 71.0 | 104.6 | 108.8 | 85.2 | 97.0 | 92.0 |
| RSD (%) | 4.1 | 2.9 | 7.9 | 13.2 | 4.0 | 9.6 | 3.0 | 5.2 | 16.7 | 20.9 |

Table S8. Mass of individual PFAS in plant roots with the amendment of PVC, PLA, and TWP (ng).

|  | ng | PFHxA | PFHpA | PFOA | PFNA | HFPO-DA | PFBS | PFHxS | PFOS | 6:2 FTSA | 8:2 FTSA |
| --- | --- | --- | --- | --- | --- | --- | --- | --- | --- | --- | --- |
| Control | AVE | - | - | - | - | - | 1.46 | - | 3.11 | 1.15 | 3.02 |
|  | SD | - | - | - | - | - | 0.23 | - | 0.08 | 0.08 | 0.55 |
| TWP-L | AVE | - | - | - | - | - | 0.94 | - | 2.80 | 1.25 | 2.11 |
|  | SD | - | - | - | - | - | 0.30 | - | 0.07 | 0.41 | 0.60 |
| TWP-M | AVE | - | - | - | - | - | 1.54 | - | 3.04 | 0.86 | 2.54 |
|  | SD | - | - | - | - | - | 0.85 | - | 0.16 | 0.10 | 0.28 |
| TWP-H | AVE | - | - | - | - | - | 2.48 | - | 4.67 | 1.50 | 4.54 |
|  | SD | - | - | - | - | - | 0.92 | - | 1.56 | 0.29 | 1.55 |
| PLA-L | AVE | - | - | - | - | - | 2.77 | - | 4.36 | 1.00 | 3.05 |
|  | SD | - | - | - | - | - | 1.89 | - | 1.56 | 0.34 | 1.31 |
| PLA-M | AVE | - | - | - | - | - | 3.30 | - | 4.27 | 1.26 | 3.26 |
|  | SD | - | - | - | - | - | 1.28 | - | 0.14 | 0.31 | 0.04 |
| PLA-H | AVE | - | - | - | - | - | 1.46 | - | 4.19 | 0.74 | 3.46 |
|  | SD | - | - | - | - | - | 0.80 | - | 0.49 | 0.21 | 0.59 |
| PVC-L | AVE | - | - | - | - | - | 1.39 | - | 2.79 | 1.00 | 1.80 |
|  | SD | - | - | - | - | - | 0.42 | - | 1.48 | 0.08 | 0.39 |
| PVC-M | AVE | - | - | - | - | - | 1.71 | - | 3.51 | 0.77 | 2.06 |
|  | SD | - | - | - | - | - | 0.16 | - | 0.77 | 0.47 | 0.45 |
| PVC-H | AVE | - | - | - | - | - | 1.39 | - | 2.18 | 0.64 | 0.79 |
|  | SD | - | - | - | - | - | 0.04 | - | 0.67 | 0.09 | 0.21 |

Table S9. Mass-normalized PFAS removal (μg PFAS/g MP) for each congener

| μg/g | PFHxA | PFHpA | PFOA | PFNA | HFPO-DA | PFBS | PFHxS | PFOS | 6:2 FTSA | 8:2 FTSA |
| --- | --- | --- | --- | --- | --- | --- | --- | --- | --- | --- |
| PVC | 28.4 | 17.1 | 19.1 | 14.6 | 2.8 | 40.4 | 19.6 | 30.7 | 18.2 | 15.0 |
| SD | 8.6 | 8.4 | 7.6 | 6.4 | 2.3 | 11.6 | 7.3 | 0.8 | 8.7 | 8.8 |
| PLA | 51.8 | 32.2 | 33.2 | 25.3 | 7.2 | 68.2 | 31.0 | 39.8 | 24.9 | 14.6 |
| SD | 4.3 | 0.8 | 3.3 | 1.2 | 3.1 | 3.8 | 2.4 | 5.5 | 1.5 | 5.2 |
| TWP | 45.9 | 30.2 | 35.2 | 46.2 | 19.1 | 62.1 | 35.2 | 76.7 | 23.6 | 26.8 |
| SD | 3.4 | 2.0 | 2.0 | 1.3 | 7.3 | 2.5 | 0.5 | 1.5 | 2.6 | 1.8 |

Table S10. Aqueous PFAS concentrations (ng/mL) after equilibration for each congener in the adsorption experiment

| ng/mL | PFHxA | PFHpA | PFOA | PFNA | HFPO-DA | PFBS | PFHxS | PFOS | 6:2 FTSA | 8:2 FTSA |
| --- | --- | --- | --- | --- | --- | --- | --- | --- | --- | --- |
| Control | 929.9 | 790.6 | 744.5 | 648.8 | 567.0 | 942.5 | 727.6 | 626.4 | 646.0 | 345.1 |
| RSD (%) | 7.8 | 4.9 | 6.8 | 5.1 | 4.1 | 10.0 | 4.8 | 9.3 | 5.7 | 21.5 |
| PVC | 788.0 | 705.0 | 648.9 | 575.8 | 553.0 | 740.6 | 629.8 | 472.8 | 554.8 | 270.1 |
| RSD (%) | 5.5 | 6.0 | 5.8 | 5.5 | 2.1 | 7.8 | 5.8 | 0.8 | 7.8 | 16.4 |
| PLA | 670.8 | 629.8 | 578.6 | 522.3 | 530.8 | 601.4 | 572.6 | 427.2 | 521.5 | 272.1 |
| RSD (%) | 3.2 | 0.6 | 2.9 | 1.1 | 2.9 | 3.2 | 2.1 | 6.4 | 1.5 | 9.6 |
| TWP | 700.5 | 639.9 | 568.4 | 417.9 | 471.4 | 632.0 | 551.6 | 243.0 | 527.9 | 210.9 |
| RSD (%) | 2.4 | 1.5 | 1.8 | 1.5 | 7.7 | 1.9 | 0.4 | 3.1 | 2.4 | 4.4 |

Note: The "control" refers to the initial PFAS concentration in the solution prior to any adsorption.

Table S11. Soil physical and chemical properties under MP exposure at 0.5% dose

|  | pH | organic matter （g/kg） | CEC （cmol/kg) | S-FDA  [μmol/(d·g)] |
| --- | --- | --- | --- | --- |
| Control | 6.60 | 15.50 | 9.36 | 204 ± 15 |
| TWP | 6.70 | 22.66 | 8.80 | 298 ± 20 |
| PLA | 6.50 | 21.50 | 9.10 | 239 ± 19 |
| PVC | 6.50 | 16.12 | 8.50 | 202 ± 29 |

Table S12. KEGG pathway enrichment analysis for the PVC-H treatment.

| Pathway_ID | First Category | Second Category | Enrich Factor | DA Score | P_adjust |
| --- | --- | --- | --- | --- | --- |
| map00240 | Metabolism | Nucleotide metabolism | 0.02 | -0.13 | 0.37 |
| map00250 | Metabolism | Amino acid metabolism | 0.04 | -0.13 | 0.28 |
| map00760 | Metabolism | Metabolism of cofactors and vitamins | 0.02 | -0.13 | 0.38 |
| map00053 | Metabolism | Carbohydrate metabolism | 0.02 | -0.14 | 0.38 |
| map00660 | Metabolism | Carbohydrate metabolism | 0.03 | -0.17 | 0.29 |
| map01250 | Metabolism | Global and overview maps | 0.00 | -0.17 | 0.71 |
| map00073 | Metabolism | Lipid metabolism | 0.04 | -0.20 | 0.28 |
| map00520 | Metabolism | Carbohydrate metabolism | 0.01 | -0.20 | 0.52 |
| map00750 | Metabolism | Metabolism of cofactors and vitamins | 0.03 | -0.20 | 0.27 |
| map00920 | Metabolism | Energy metabolism | 0.03 | -0.20 | 0.29 |
| map00040 | Metabolism | Carbohydrate metabolism | 0.02 | -0.25 | 0.37 |
| map04075 | Environmental Information Processing | Signal transduction | 0.08 | -0.25 | 0.17 |
| map00430 | Metabolism | Metabolism of other amino acids | 0.04 | -0.33 | 0.26 |
| map00710 | Metabolism | Energy metabolism | 0.04 | -0.33 | 0.26 |
| map00190 | Metabolism | Energy metabolism | 0.06 | -1.00 | 0.20 |
| map00523 | Metabolism | Metabolism of terpenoids and polyketides | 0.02 | -1.00 | 0.37 |
| map00470 | Metabolism | Metabolism of other amino acids | 0.01 | 0.07 | 0.38 |
| map00130 | Metabolism | Metabolism of cofactors and vitamins | 0.01 | 0.10 | 0.38 |
| map00260 | Metabolism | Amino acid metabolism | 0.02 | 0.10 | 0.35 |
| map00460 | Metabolism | Metabolism of other amino acids | 0.02 | 0.11 | 0.34 |
| map01040 | Metabolism | Lipid metabolism | 0.01 | 0.14 | 0.39 |
| map00901 | Metabolism | Biosynthesis of other secondary metabolites | 0.01 | 0.50 | 0.40 |
| map00630 | Metabolism | Carbohydrate metabolism | 0.03 | -0.18 | 0.16 |
| map00310 | Metabolism | Amino acid metabolism | 0.04 | -0.25 | 0.13 |
| map00280 | Metabolism | Amino acid metabolism | 0.05 | -0.33 | 0.11 |
| map00650 | Metabolism | Carbohydrate metabolism | 0.04 | -0.40 | 0.11 |
| map00020 | Metabolism | Carbohydrate metabolism | 0.10 | -0.50 | 0.04 |
| map00620 | Metabolism | Carbohydrate metabolism | 0.06 | -1.00 | 0.08 |
| map00592 | Metabolism | Lipid metabolism | 0.05 | 0.00 | 0.11 |
| map02010 | Environmental Information Processing | Membrane transport | 0.01 | 0.00 | 0.29 |
| map00970 | Genetic Information Processing | Translation | 0.04 | 0.12 | 0.12 |
| map00380 | Metabolism | Amino acid metabolism | 0.02 | 0.13 | 0.20 |
| map00966 | Metabolism | Biosynthesis of other secondary metabolites | 0.03 | 0.14 | 0.18 |
| map00400 | Metabolism | Amino acid metabolism | 0.06 | 0.17 | 0.09 |
| map00960 | Metabolism | Biosynthesis of other secondary metabolites | 0.03 | 0.17 | 0.18 |
| map00950 | Metabolism | Biosynthesis of other secondary metabolites | 0.02 | 0.25 | 0.29 |
| map00640 | Metabolism | Carbohydrate metabolism | 0.07 | -0.75 | 0.01 |
| map01240 | Metabolism | Global and overview maps | 0.01 | 0.02 | 0.37 |
| map00996 | Metabolism | Biosynthesis of other secondary metabolites | 0.04 | 0.27 | 0.06 |
| map00360 | Metabolism | Amino acid metabolism | 0.08 | 0.00 | 0.00 |
| map00940 | Metabolism | Biosynthesis of other secondary metabolites | 0.07 | 0.09 | 0.00 |
| map00350 | Metabolism | Amino acid metabolism | 0.06 | -0.06 | 0.00 |
| map00999 | Metabolism | Biosynthesis of other secondary metabolites | 0.03 | 0.04 | 0.02 |

Table S13. KEGG pathway enrichment analysis for the PLA-H treatment.

| Pathway_ID | First Category | Second Category | Enrich Factor | DA Score | P_adjust |
| --- | --- | --- | --- | --- | --- |
| map02010 | Environmental Information Processing | Membrane transport | 0.01 | -0.04 | 0.70 |
| map00970 | Genetic Information Processing | Translation | 0.02 | -0.06 | 0.55 |
| map00470 | Metabolism | Metabolism of other amino acids | 0.01 | -0.07 | 0.53 |
| map00270 | Metabolism | Amino acid metabolism | 0.01 | -0.08 | 0.53 |
| map00630 | Metabolism | Carbohydrate metabolism | 0.02 | -0.09 | 0.55 |
| map00260 | Metabolism | Amino acid metabolism | 0.02 | -0.10 | 0.55 |
| map00410 | Metabolism | Metabolism of other amino acids | 0.03 | -0.11 | 0.56 |
| map00460 | Metabolism | Metabolism of other amino acids | 0.02 | -0.11 | 0.56 |
| map00240 | Metabolism | Nucleotide metabolism | 0.02 | -0.13 | 0.55 |
| map00310 | Metabolism | Amino acid metabolism | 0.02 | -0.13 | 0.54 |
| map00760 | Metabolism | Metabolism of cofactors and vitamins | 0.02 | -0.13 | 0.55 |
| map00944 | Metabolism | Biosynthesis of other secondary metabolites | 0.02 | -0.13 | 0.56 |
| map00053 | Metabolism | Carbohydrate metabolism | 0.02 | -0.14 | 0.54 |
| map00480 | Metabolism | Metabolism of other amino acids | 0.03 | -0.14 | 0.52 |
| map00340 | Metabolism | Amino acid metabolism | 0.02 | -0.17 | 0.56 |
| map00660 | Metabolism | Carbohydrate metabolism | 0.03 | -0.17 | 0.51 |
| map00590 | Metabolism | Lipid metabolism | 0.01 | -0.20 | 0.53 |
| map00750 | Metabolism | Metabolism of cofactors and vitamins | 0.03 | -0.20 | 0.58 |
| map00920 | Metabolism | Energy metabolism | 0.03 | -0.20 | 0.50 |
| map00020 | Metabolism | Carbohydrate metabolism | 0.05 | -0.25 | 0.53 |
| map00040 | Metabolism | Carbohydrate metabolism | 0.02 | -0.25 | 0.53 |
| map00332 | Metabolism | Biosynthesis of other secondary metabolites | 0.03 | -0.25 | 0.56 |
| map00740 | Metabolism | Metabolism of cofactors and vitamins | 0.04 | -0.25 | 0.55 |
| map00908 | Metabolism | Metabolism of terpenoids and polyketides | 0.03 | -0.25 | 0.51 |
| map00945 | Metabolism | Biosynthesis of other secondary metabolites | 0.04 | -0.25 | 0.53 |
| map00710 | Metabolism | Energy metabolism | 0.04 | -0.33 | 0.56 |
| map00730 | Metabolism | Metabolism of cofactors and vitamins | 0.03 | -0.33 | 0.58 |
| map00620 | Metabolism | Carbohydrate metabolism | 0.03 | -0.50 | 0.56 |
| map04122 | Genetic Information Processing | Folding, sorting and degradation | 0.09 | -0.50 | 0.35 |
| map00942 | Metabolism | Biosynthesis of other secondary metabolites | 0.02 | -1.00 | 0.54 |
| map00380 | Metabolism | Amino acid metabolism | 0.01 | 0.07 | 0.52 |
| map00966 | Metabolism | Biosynthesis of other secondary metabolites | 0.01 | 0.07 | 0.53 |
| map00960 | Metabolism | Biosynthesis of other secondary metabolites | 0.01 | 0.08 | 0.53 |
| map00996 | Metabolism | Biosynthesis of other secondary metabolites | 0.01 | 0.09 | 0.54 |
| map00524 | Metabolism | Biosynthesis of other secondary metabolites | 0.01 | 0.50 | 0.53 |
| map00770 | Metabolism | Metabolism of cofactors and vitamins | 0.07 | -0.18 | 0.14 |
| map00591 | Metabolism | Lipid metabolism | 0.07 | -0.20 | 0.18 |
| map00430 | Metabolism | Metabolism of other amino acids | 0.08 | -0.67 | 0.16 |
| map00360 | Metabolism | Amino acid metabolism | 0.04 | 0.00 | 0.28 |
| map00999 | Metabolism | Biosynthesis of other secondary metabolites | 0.02 | -0.11 | 0.48 |
| map00941 | Metabolism | Biosynthesis of other secondary metabolites | 0.04 | -0.27 | 0.16 |
| map01232 | Metabolism | Global and overview maps | 0.05 | -0.27 | 0.15 |
| map01040 | Metabolism | Lipid metabolism | 0.04 | 0.14 | 0.16 |
| map00940 | Metabolism | Biosynthesis of other secondary metabolites | 0.07 | -0.17 | 0.03 |
| map00592 | Metabolism | Lipid metabolism | 0.11 | -0.18 | 0.00 |
| map00230 | Metabolism | Nucleotide metabolism | 0.05 | -0.45 | 0.02 |
| map01240 | Metabolism | Global and overview maps | 0.02 | -0.14 | 0.27 |

Table S14. KEGG pathway enrichment analysis for the TWP-H treatment.

| Pathway_ID | First Category | Second Category | Enrich Factor | DA Score | P_adjust |
| --- | --- | --- | --- | --- | --- |
| map00970 | Genetic Information Processing | Translation | 0.02 | -0.06 | 0.41 |
| map00470 | Metabolism | Metabolism of other amino acids | 0.01 | -0.07 | 0.41 |
| map00966 | Metabolism | Biosynthesis of other secondary metabolites | 0.01 | -0.07 | 0.38 |
| map00760 | Metabolism | Metabolism of cofactors and vitamins | 0.02 | -0.13 | 0.40 |
| map00480 | Metabolism | Metabolism of other amino acids | 0.03 | -0.14 | 0.35 |
| map01040 | Metabolism | Lipid metabolism | 0.01 | -0.14 | 0.40 |
| map00073 | Metabolism | Lipid metabolism | 0.04 | -0.20 | 0.33 |
| map00590 | Metabolism | Lipid metabolism | 0.01 | -0.20 | 0.37 |
| map00750 | Metabolism | Metabolism of cofactors and vitamins | 0.03 | -0.20 | 0.32 |
| map00740 | Metabolism | Metabolism of cofactors and vitamins | 0.04 | -0.25 | 0.32 |
| map00940 | Metabolism | Biosynthesis of other secondary metabolites | 0.02 | 0.04 | 0.40 |
| map00996 | Metabolism | Biosynthesis of other secondary metabolites | 0.01 | 0.09 | 0.39 |
| map00130 | Metabolism | Metabolism of cofactors and vitamins | 0.01 | 0.10 | 0.40 |
| map00790 | Metabolism | Metabolism of cofactors and vitamins | 0.02 | 0.14 | 0.40 |
| map02010 | Environmental Information Processing | Membrane transport | 0.01 | -0.09 | 0.36 |
| map00230 | Metabolism | Nucleotide metabolism | 0.02 | -0.18 | 0.32 |
| map01232 | Metabolism | Global and overview maps | 0.03 | -0.18 | 0.15 |
| map00360 | Metabolism | Amino acid metabolism | 0.04 | 0.00 | 0.13 |
| map00908 | Metabolism | Metabolism of terpenoids and polyketides | 0.05 | 0.00 | 0.10 |
| map00999 | Metabolism | Biosynthesis of other secondary metabolites | 0.01 | 0.00 | 0.35 |
| map00350 | Metabolism | Amino acid metabolism | 0.03 | 0.13 | 0.23 |
| map00270 | Metabolism | Amino acid metabolism | 0.04 | -0.08 | 0.05 |
| map00591 | Metabolism | Lipid metabolism | 0.14 | -0.40 | 0.00 |
| map01240 | Metabolism | Global and overview maps | 0.02 | -0.09 | 0.06 |
| map00592 | Metabolism | Lipid metabolism | 0.16 | -0.41 | 0.00 |

Table S15. Top 30 metabolites with VIP > 1, *p* < 0.05 in plants shoots exposed to PVC-H

| Metabolite | VIP | *p* | Regulate |
| --- | --- | --- | --- |
| Taurocholic acid | 3.92 | 0.029 | down |
| Ser-Phe-Val-Lys | 3.56 | 0.016 | down |
| P-Menthane-3,8-diol | 2.84 | 0.022 | down |
| Pantoyllactone glucoside | 2.73 | 0.022 | down |
| (3R,6'Z)-3,4-Dihydro-8-hydroxy-3-(6-pentadecenyl)-1H-2-benzopyran-1-one | 2.63 | 0.031 | down |
| D-Glucuronic acid 1-phosphate | 2.62 | 0.010 | down |
| 3,7-dihydropurin-6-one | 2.53 | 0.026 | down |
| L-xylo-hex-3-ulono-1,4-lactone | 2.48 | 0.015 | down |
| Fabianine | 2.40 | 0.036 | down |
| 4,7-Dimethyl-1-tetralone | 2.36 | 0.011 | down |
| Ocophyllal B | 2.29 | 0.005 | down |
| Arteincultone | 2.22 | 0.006 | down |
| Albocycline M-1 | 2.22 | 0.040 | down |
| (1S,2R,4R)-p-Menth-8-ene-2,10-diol 2-glucoside | 2.20 | 0.005 | down |
| Indole-3-acetylglutamic acid | 2.15 | 0.029 | up |
| Methyl (10E,12Z,15Z)-9-hydroxyoctadeca-10,12,15-trienoate | 2.09 | 0.047 | down |
| Lactucin | 2.07 | 0.026 | down |
| 1,3-diphenylurea | 2.06 | 0.024 | up |
| Trans-Cinnamic acid | 2.04 | 0.003 | up |
| (+)-4,5-Didehydrojasmonic acid | 2.03 | 0.014 | down |
| Trichotriol | 1.99 | 0.031 | down |
| L-malic acid | 1.97 | 0.000 | down |
| Kobusimin B | 1.95 | 0.042 | down |
| Normetanephrine | 1.94 | 0.004 | up |
| 1.3-dilinolenin | 1.90 | 0.003 | up |
| Methylmalonic acid | 1.83 | 0.018 | down |
| Albocycline | 1.82 | 0.010 | down |
| D-(+)-Tryptophan | 1.81 | 0.008 | up |
| Tyramine | 1.75 | 0.008 | up |
| Tryptophol | 1.75 | 0.015 | up |

Table S16. Top 30 metabolites with VIP > 1, *p* < 0.05 in plants shoots exposed to PLA-H

| Metabolite | VIP | *p* | Regulate |
| --- | --- | --- | --- |
| Taurocholic acid | 3.60 | 0.029 | down |
| Patuletin 3-(2''-apiosyl-[2'''-feruloylgentiobioside]) | 3.23 | 0.039 | down |
| (-)-Epigallocatechin 3-cinnamate | 3.16 | 0.015 | down |
| Ser-Phe-Val-Lys | 3.15 | 0.021 | down |
| Vanillic acid 4-O-glucuronide | 2.95 | 0.016 | down |
| P-Menthane-3,8-diol | 2.95 | 0.000 | down |
| Lesquerolic acid | 2.70 | 0.023 | up |
| Rumphellolide F | 2.66 | 0.002 | down |
| Linalool (8-hydroxydihydro-) | 2.56 | 0.031 | up |
| 6-Methoxy-7-[3,4,5-trihydroxy-6-(hydroxymethyl)oxan-2-yl]oxychromen-2-one | 2.55 | 0.010 | down |
| Ganoderic acid f | 2.49 | 0.022 | up |
| Cryptochlorogenic acid | 2.33 | 0.034 | down |
| 4-Caffeoylquinic acid | 2.32 | 0.038 | down |
| Phaseolic acid | 2.31 | 0.041 | down |
| 5-Hydroxyferulate | 2.22 | 0.033 | down |
| Amabiline | 2.17 | 0.040 | down |
| 5,7-dihydroxy-4-methylphthalide | 2.16 | 0.040 | down |
| 3-hydroxycoumarin | 2.16 | 0.035 | down |
| 5,7-Dihydroxycoumarin | 2.14 | 0.045 | down |
| Daphnetin | 2.09 | 0.025 | down |
| 5'-O-beta-D-Glucosylpyridoxine | 2.05 | 0.007 | down |
| Guanosine 5'-monophosphate | 2.05 | 0.013 | down |
| Pantoyllactone glucoside | 2.04 | 0.009 | down |
| Alpha-Campholonic acid | 2.03 | 0.005 | down |
| Neomycin | 2.03 | 0.027 | up |
| Arachidonic acid | 2.01 | 0.007 | down |
| Methyl (10E,12Z,15Z)-9-hydroxyoctadeca-10,12,15-trienoate | 2.01 | 0.050 | down |
| Adenosine 3'-monophosphate | 2.00 | 0.004 | down |
| Piceol | 2.00 | 0.036 | down |
| Caffeic acid | 1.99 | 0.009 | down |

Table S17. Top 30 metabolites with VIP > 1, *p* < 0.05 in plants shoots exposed to TWP-H

| Metabolite | VIP | *p* | Regulate |
| --- | --- | --- | --- |
| Rumphellolide F | 3.19 | 0.009 | down |
| Monomenthyl succinate | 2.78 | 0.007 | up |
| P-Menthane-3,8-diol | 2.61 | 0.008 | down |
| 6alpha-Carissanol | 2.61 | 0.050 | down |
| Emedastine | 2.59 | 0.012 | down |
| Pentyl glucosinolate | 2.55 | 0.004 | down |
| 3-Deoxyestradiol | 2.52 | 0.002 | down |
| Zerumbone | 2.51 | 0.010 | down |
| Porrigenic acid | 2.49 | 0.004 | down |
| 3alpha-Methoxy-desoxo-achalensolide | 2.43 | 0.008 | down |
| Albocycline | 2.38 | 0.014 | down |
| Cis-Hexadec-11-en-7,9-diynoic acid | 2.36 | 0.003 | down |
| Fabianine | 2.35 | 0.036 | down |
| 12-Oxo-2,3-dinor-10,15-phytodienoic acid | 2.34 | 0.003 | down |
| Ganoderic acid f | 2.32 | 0.018 | up |
| 9-OxoOTrE | 2.32 | 0.003 | down |
| (Z)-Octadec-12-ene-8,10-diynoic acid | 2.29 | 0.004 | down |
| Andrographiside | 2.27 | 0.001 | down |
| 19-Hydroxycinnzeylanol 19-glucoside | 2.26 | 0.018 | down |
| Achaetolide | 2.25 | 0.008 | down |
| Methyl 2-methyl-6-(4-methyl-2-oxocyclohex-3-en-1-yl)hept-2-enoate | 2.22 | 0.006 | down |
| 12,13-trans-Epoxy-9-oxo-10E,15Z-octadecadienoic acid | 2.19 | 0.027 | down |
| 4,7-Dimethyl-1-tetralone | 2.16 | 0.025 | down |
| Isoboonein | 2.16 | 0.002 | down |
| 9,12-octadecadiynoic acid | 2.15 | 0.021 | down |
| Brefeldin a | 2.15 | 0.046 | down |
| Ile-Ile-Ala | 2.14 | 0.014 | down |
| 3-Hydroxy-alpha-ionone | 2.14 | 0.002 | down |
| Ieodomycin D | 2.13 | 0.005 | down |
| 13-OxoODE | 2.12 | 0.024 | down |


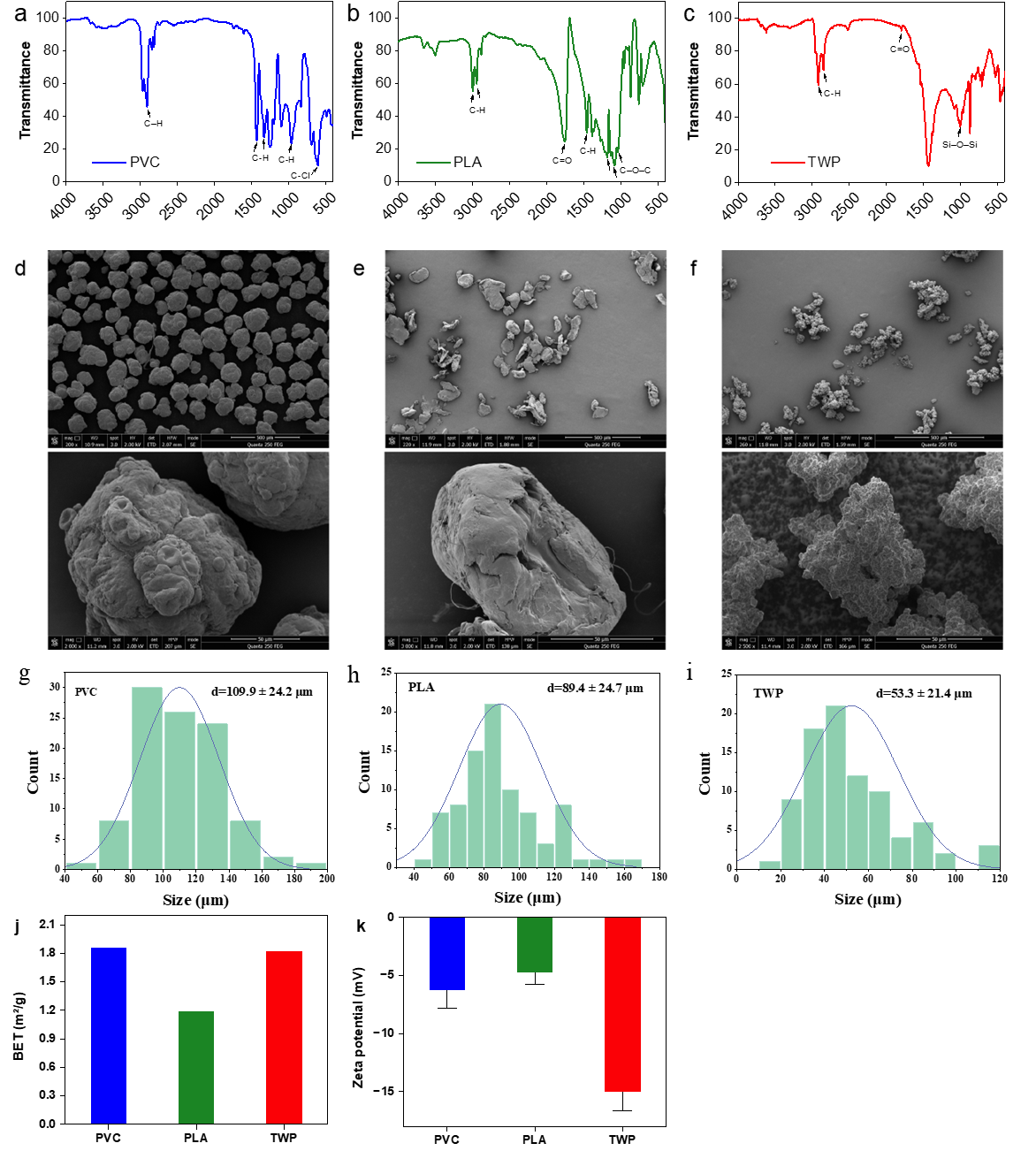


Fig. S1. Characterization of MPs. Fourier infrared Spectrum of PVC, PLA, and TWP (a-c); SEM of PVC, PLA, and TWP (d-f); particle size distribution of PVC, PLA, and TWP (g-i); specific surface area measured by BET (j), and zeta potential (k) of PVC, PLA, and TWP.


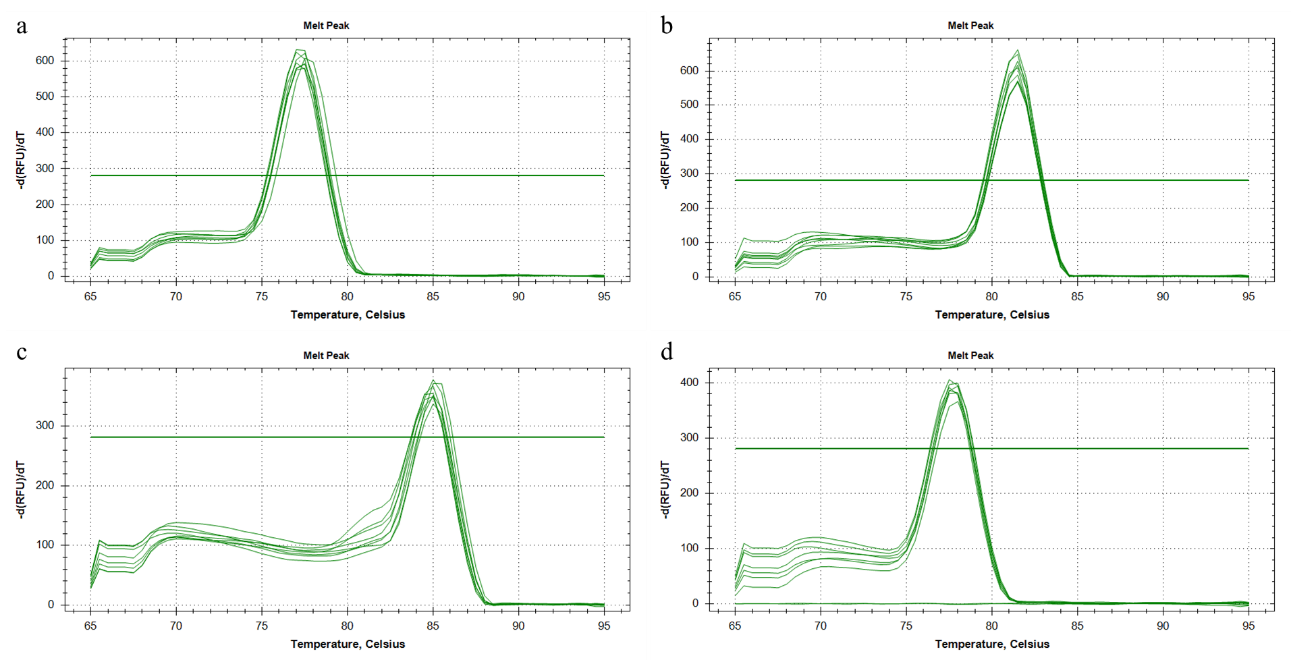


Fig. S2 . Representative melt-curve profiles for qPCR amplifications of target genes: (a) *PIP1-1*, (b) *TIP1-1*, (c) *TIP1-2*, and (d) *NIP5-1*

Fig. S3. Mass balance of 10 PFASs in the whole plant exposure experiments with the treatments of MPs

Fig. S4. Bioconcentration of individual PFAS in plant shoots with the amendment of PVC (A), PLA (B), and TWP (C) at the dose of 0.01%, 0.05%, and 0.5%. Significant differences from the control are indicated by *p* < 0.05 (*) and *p* < 0.01 (**), respectively.


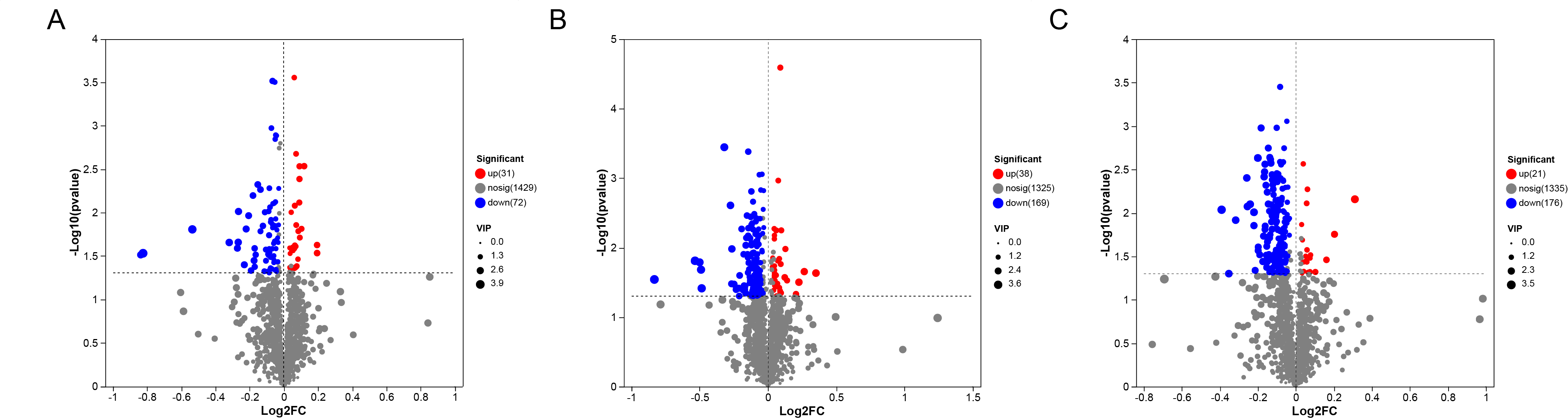


Fig. S5. Volcano maps showing differential metabolites in plant shoots between control and PVC (A), PLA (B), and TWP (C)


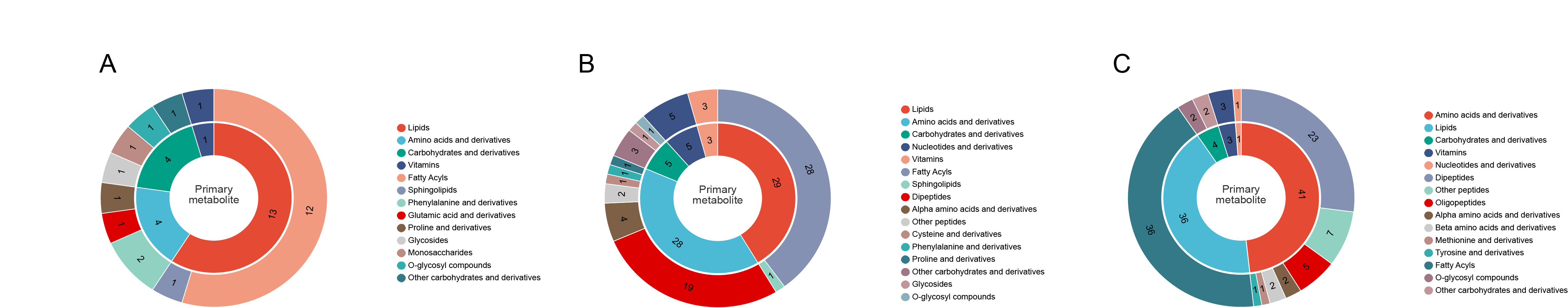


Fig. S6. Primary metabolites in plant shoots with the amendment of PVC (A), PLA (B), and TWP (C).


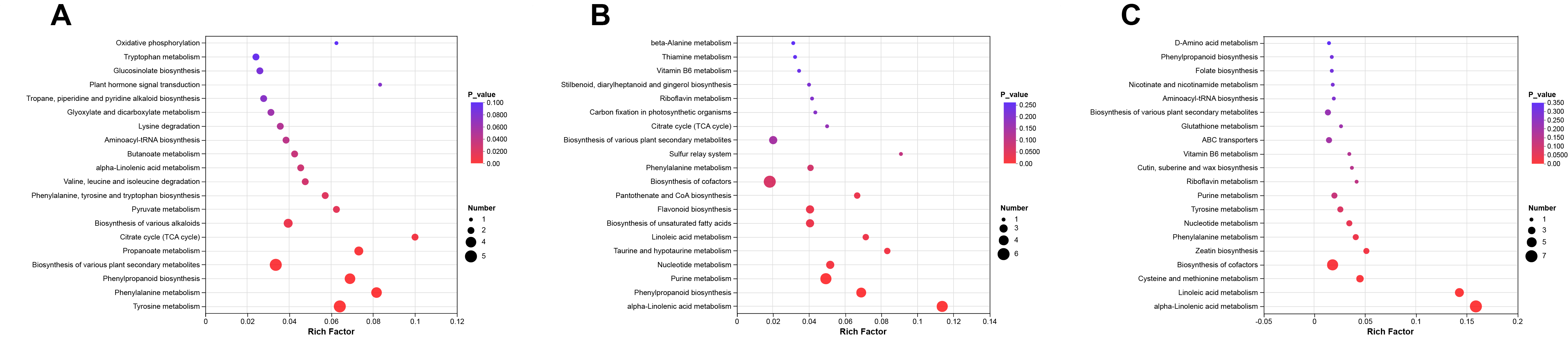


Fig. S7. KEGG pathways analysis of plant shoots with the amendment of PVC (A), PLA (B), and TWP (C).

**References**

(1) C. V. A. Scordo, L. Checchini, L. Renai, S. Orlandini, M. C. Bruzzoniti, D. Fibbi*, et al.* Optimization and validation of a method based on QuEChERS extraction and liquid chromatographic-tandem mass spectrometric analysis for the determination of perfluoroalkyl acids in strawberry and olive fruits, as model crops with different matrix characteristics. *J. Chromatogr. A 1621* (2020) 461038 <https://doi.org/10.1016/j.chroma.2020.461038>

(2) G. Dierkes, T. Lauschke, S. Becher, H. Schumacher, C. Foeldi,T. Ternes. Quantification of microplastics in environmental samples via pressurized liquid extraction and pyrolysis-gas chromatography. *Anal. Bioanal. Chem. 411* (2019) 6959-6968 <https://doi.org/10.1007/s00216-019-02066-9>

(3) W. F. Li, R. Wufuer, J. Duo, S. Z. Wang, Y. M. Luo, D. Y. Zhang*, et al.* Microplastics in agricultural soils: Extraction and characterization after different periods of polythene film mulching in an arid region. *Sci. Total Environ. 749* (2020) 141420 <https://doi.org/10.1016/j.scitotenv.2020.141420>

(4) S. L. Zhang, X. M. Yang, H. Gertsen, P. Peters, T. Salánki,V. Geissen. A simple method for the extraction and identification of light density microplastics from soil. *Sci. Total Environ. 616* (2018) 1056-1065 <https://doi.org/10.1016/j.scitotenv.2017.10.213>

(5) G. Colombini, C. Rumpel, S. Houot, P. Biron,M. F. Dignac. A long-term field experiment confirms the necessity of improving biowaste sorting to decrease coarse microplastic inputs in compost amended soils. *Environ. Pollut. 315* (2022) 120369 <https://doi.org/10.1016/j.envpol.2022.120369>

(6) Jes Vollertsen,A. A. Hansen. Microplastic in Danish wastewater: Sources, occurrences and fate; The Danish Environmental Protection Agency, 2017.

(7) F. Corradini, P. Meza, R. Eguiluz, F. Casado, E. Huerta-Lwanga,V. Geissen. Evidence of microplastic accumulation in agricultural soils from sewage sludge disposal. *Sci. Total Environ. 671* (2019) 411-420 <https://doi.org/10.1016/j.scitotenv.2019.03.368>

(8) J. Crossman, R. R. Hurley, M. Futter,L. Nizzetto. Transfer and transport of microplastics from biosolids to agricultural soils and the wider environment. *Sci. Total Environ. 724* (2020) 138334 <https://doi.org/10.1016/j.scitotenv.2020.138334>

(9) S. Fuller,A. Gautam. A Procedure for Measuring Microplastics using Pressurized Fluid Extraction. *Environ. Sci. Technol. 50* (2016) 5774-5780 <https://doi.org/10.1021/acs.est.6b00816>

(10) J. M. Panko, J. Chu, M. L. Kreider,K. M. Unice. Measurement of airborne concentrations of tire and road wear particles in urban and rural areas of France, Japan, and the United States. *Atmos. Environ. 72* (2013) 192-199 <https://doi.org/10.1016/j.atmosenv.2013.01.040>

(11) J. Ding, M. Lv, D. Zhu, E. F. Leifheit, Q. L. Chen, Y. Q. Wang*, et al.* Tire wear particles: An emerging threat to soil health. *Crit. Rev. Environ. Sci. Technol. 53* (2023) 239-257 <https://doi.org/10.1080/10643389.2022.2047581>
